# Divergent trajectories of cellular bioenergetics, intermediary metabolism and systemic redox status in survivors and non-survivors of critical illness

**DOI:** 10.1016/j.redox.2021.101907

**Published:** 2021-02-20

**Authors:** Helen T. McKenna, Katie A. O'Brien, Bernadette O. Fernandez, Magdalena Minnion, Adam Tod, Ben D. McNally, James A. West, Julian L. Griffin, Michael P. Grocott, Michael G. Mythen, Martin Feelisch, Andrew J. Murray, Daniel S. Martin

**Affiliations:** a Division of Surgery and Interventional Science, University College London, Royal Free Hospital, London, NW3 2QG, UK; b Intensive Care Unit, Royal Free Hospital, London, NW3 2QG, UK; c Department of Physiology, Development and Neuroscience, University of Cambridge, Cambridge, CB2 3EG, UK; d Clinical & Experimental Sciences, Faculty of Medicine, University of Southampton, Southampton, SO16 6YD, UK; e Department of Biochemistry and the Cambridge Systems Biology Centre, University of Cambridge, CB2 1GA, UK; f Cambridge Institute of Therapeutic Immunology and Infectious Disease, Department of Medicine, Jeffrey Cheah Biomedical Centre, University of Cambridge, CB2 0RE, UK; g Section of Biomolecular Medicine, Department of Digestion, Metabolism and Reproduction, Imperial College London, SW7 2AZ, UK; h Anaesthesia Perioperative and Critical Care Research Group, Southampton National Institute of Health Research Biomedical Research Centre, University Hospital Southampton, SO16 6YD, UK; i University College London Hospitals and Great Ormond Street, National Institute of Health Research Biomedical Research Centres, London, WC1N 1EH, UK; j Peninsula Medical School, University of Plymouth, John Bull Building, Derriford, Plymouth, PL6 8BU, UK

**Keywords:** Critical illness, Stress physiology, Energy metabolism, Mitochondria, Redox signaling, Oxidative stress

## Abstract

**Background:**

Numerous pathologies result in multiple-organ failure, which is thought to be a direct consequence of compromised cellular bioenergetic status. Neither the nature of this phenotype nor its relevance to survival are well understood, limiting the efficacy of modern life-support.

**Methods:**

To explore the hypothesis that survival from critical illness relates to changes in cellular bioenergetics, we combined assessment of mitochondrial respiration with metabolomic, lipidomic and redox profiling in skeletal muscle and blood, at multiple timepoints, in 21 critically ill patients and 12 reference patients.

**Results:**

We demonstrate an end-organ cellular phenotype in critical illness, characterized by preserved total energetic capacity, greater coupling efficiency and selectively lower capacity for complex I and fatty acid oxidation (FAO)-supported respiration in skeletal muscle, compared to health. In survivors, complex I capacity at 48 h was 27% lower than in non-survivors (p = 0.01), but tended to increase by day 7, with no such recovery observed in non-survivors. By day 7, survivors’ FAO enzyme activity was double that of non-survivors (p = 0.048), in whom plasma triacylglycerol accumulated. Increases in both cellular oxidative stress and reductive drive were evident in early critical illness compared to health. Initially, non-survivors demonstrated greater plasma total antioxidant capacity but ultimately higher lipid peroxidation compared to survivors. These alterations were mirrored by greater levels of circulating total free thiol and nitrosated species, consistent with greater reductive stress and vascular inflammation, in non-survivors compared to survivors. In contrast, no clear differences in systemic inflammatory markers were observed between the two groups.

**Conclusion:**

Critical illness is associated with rapid, specific and coordinated alterations in the cellular respiratory machinery, intermediary metabolism and redox response, with different trajectories in survivors and non-survivors. Unravelling the cellular and molecular foundation of human resilience may enable the development of more effective life-support strategies.

## Graphical abstract

**Figure:**
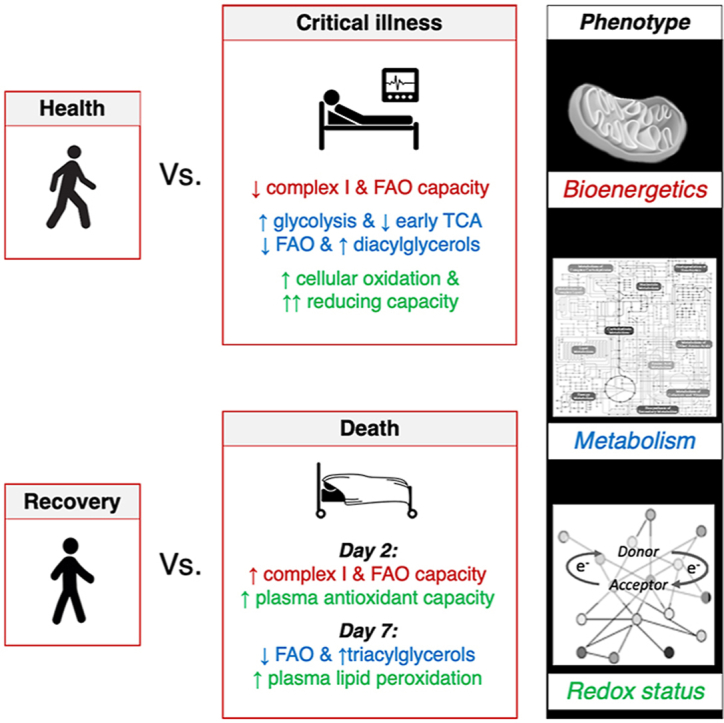

## 1 Introduction

Critical illness is poorly defined but generally refers to a condition in which acute organ dysfunction, involving two or more organs, has occurred to the degree that supportive intervention is required on an intensive care unit (ICU) [1]. Many different pathologies may lead to this heterogeneous state. No definite explanation for multiple-organ failure has been found, but it generally involves tissue injury (from trauma, infection, or other inflammatory cause), triggering a systemic humoral and inflammatory response, with subsequent alterations to macro- and microcirculatory blood flow and tissue oxygen demand [2]. It is associated with high short-term mortality (approximately 35–65%) even with provision of artificial organ support [3,4].

More than five million patients are admitted to an ICU each year in the USA and the cost of hospital admissions to ICUs accounts for almost half of all hospital expenditure [5]. The recent COVID-19 pandemic has demonstrated the devastating impact of critical illness on a global scale. It also highlights the pressing need to develop more sophisticated strategies to support organ function in this, still poorly understood, life-threatening state. The impact of critical illness extends far beyond the ICU, with many survivors suffering long-term physical and psychological impairment for years following discharge [6], creating a major economic burden, on top of untold suffering of the individuals and their families. Despite clear statements that what we require is a better understanding of the basic cellular mechanisms that underlie critical illness [7], very little progress has been made in the last decade [8].

Cellular bioenergetic failure is purported to underlie organ dysfunction in critical illness [9,10], therefore the majority of supportive therapy has traditionally focused on the augmentation of oxygen transport and macrocirculatory blood flow, with the avowed aim of promoting cellular ATP production by mitochondrial oxidative phosphorylation. However, clinical trials have failed to demonstrate that this approach leads to a tangible improvement in patient survival [11]. An alternative hypothesis is that recovery from multiple-organ failure depends upon the ability of cells to adapt to the pathophysiological stress generated by critical illness, which imposes extreme deviations in multiple factors influencing cellular bioenergetic demand and supply. Cellular adaptation to analogous forms of bioenergetic stress, such as that imposed by environmental hypoxia, has been demonstrated in studies of acclimatizing humans [12] and in human cells ex vivo [13]. Cellular accommodation to the specific bioenergetic demands of critical illness would require a physiological switch to enable adequate adaptation at multiple levels of biological organization. Reduction-oxidation (redox) reactions are thought to be central to the signaling and synchronization of processes within cells and between different body compartments to meet altered bioenergetic and metabolic demands in response to stress [14]. A complex series of cascading redox interactions between different bioactive species, across multiple compartments within the body, represents a plausible candidate mechanism for the integration of critical illness inflammatory and stress signaling to coordinate putative adaptations in cellular bioenergetics.

Without reliable markers of cellular performance in the ICU setting, investigations of the bioenergetic response to critical illness have been limited and the results inconsistent. Generally, skeletal muscle physiology has been used as a surrogate for other, arguably more essential, organs, due to the relatively low risk of harm from obtaining tissue samples in critically unwell patients. Lower intramuscular ATP concentrations have been measured in patients with sepsis-induced multiple organ failure compared to patients undergoing elective surgery [15], and in a heterogenous group of critically ill patients compared to ambulant patients with respiratory disease [16]. However, supranormal intramuscular ATP concentrations have also been described during critical illness, at least in patients who went on to survive [17]. As a surrogate marker of bioenergetic capacity, tissue ATP concentrations are limited by the fact that they reflect the steady-state value of both production and consumption, which are highly regulated and buffered by phosphocreatine [18]. Systemic redox alterations, as evidenced by increased concentrations of oxidized macromolecules, have been implicated in the genesis and perpetuation of sepsis, a common cause of critical illness, and oxidative stress has also been linked to changes in mitochondrial function [19]. To determine whether bioenergetic capacity truly represents a viable target to promote survival, a more comprehensive understanding is required of how these complex cellular processes operate in humans, in the real-world context of life-threatening stress.

We hypothesized that patients with critical illness (defined by multiple-organ failure) would exhibit a distinct cellular phenotype, encompassing bioenergetic, metabolic and redox function, which differs from health, and that specific changes in this phenotype would distinguish eventual survivors from non-survivors. To this end, we combined direct assessment of mitochondrial respiration with contemporaneous characterization of relevant metabolic pathways (including glycolysis, the tricarboxylic acid (TCA) cycle, fatty acid oxidation (FAO), and amino acid metabolism) and redox status in skeletal muscle from patients with acute onset multiple-organ failure and from a healthy reference group. We also investigated the relationship between cellular processes and the systemic compartment by concomitant assessment of plasma lipidomic and redox profiles with oxidative stress markers in erythrocytes.

## 2 Results

### 2.1 Integrated bioenergetic, metabolic and redox phenotyping in patients with critical illness

**Fig. 1:**
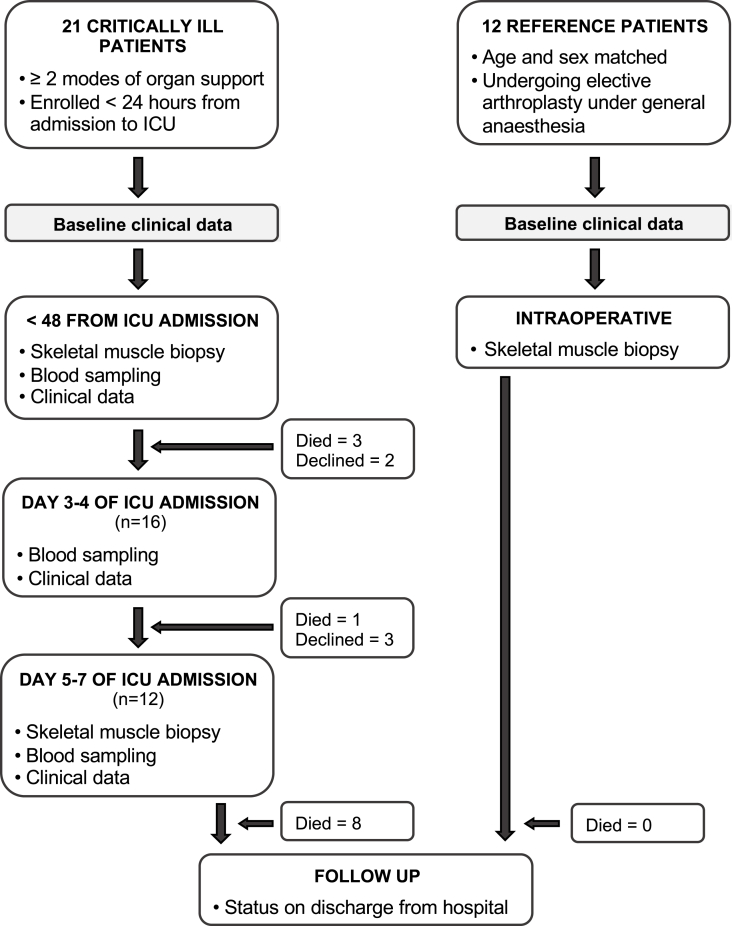
Study schedule for tissue and blood sample collection from critically ill patients and the healthy reference cohort.

We recruited adult ICU patients with acute onset, severe physiological derangement (Table 1), from any primary cause (Table 2), requiring at least two forms of artificial organ support (n = 21). The study protocol is outlined in Fig. 1. A vastus lateralis muscle biopsy was performed within 48 h following admission to the ICU and repeated, when possible, at day 5–7 (n = 12). Blood was sampled from arterial and central venous catheters at the same timepoints, with an additional sample taken at day 3–4. Detailed clinical data were also collected at baseline, day 3–4 and day 5–7 and are shown in Supplementary Table 1. Muscle biopsies and demographic and basic clinical data were taken from a healthy reference group of age and sex-matched patients undergoing elective hip surgery under general anesthesia (n = 12, Table 1). In the cohort of patients with critical illness, the severity of organ failure at enrolment was high, according to APACHE II (mean 28; s.d. 7) and SOFA scores (mean 13; s.d. 3), and 12 patients died prior to hospital discharge. Non-survivors were, on average, 15 years older than survivors, with a greater severity of illness according to APACHE II score (Supplementary Table 2). No patient was hypoxemic, as judged by arterial partial pressure of oxygen, peripheral arterial oxygen saturation or calculated arterial oxygen content (Supplementary Table 2).

**Table 1:** Baseline characteristics of critically ill and healthy reference cohorts.

Participant characteristics	Critically ill (<48 h ICU admission) (n = 21)	Reference group (n = 12)
Age, y mean (95% CI)	61.4 (54.4–68.4)	59.5 (49.8–69.2)
Female sex, n (%)	8 [38]	7 [58]
Height, m median (IQR)	1.75 (1.66–1.77)	1.73 (1.67–1.77)
Weight, kg median (IQR)	70.6 (63.8–90)	78.0 (65.7–96.8)
BMI, kg/m^2^ median (IQR)	24.2 (22.0–29.5)	25.4 (22.3–31.9)
Organ insufficiency prior to admission, n (%)	3 [14]	0 (0)
Independent prior to hospital admission, n (%)	20 (95)	12 (100)
Co-morbidities n (%)		
Hypertension	11 [54]	1 [8]
Ischemic heart disease/arrythmia	6 [29]	1 [8]
Respiratory	6 [29]	2 [17]
Diabetes	4 [19]	0 (0)
Chronic renal impairment	3 [14]	0 (0)
Mechanically ventilated n (%)	21 (100)	12 (100)
Intravenous antibiotic therapy n (%)	19 (90)	12 (100)
Propofol administered n (%)	11 [52]	12 (100)
Muscle relaxant within previous 24 h n (%)	9 [43]	12 [12]
Nutrition within previous 6 h n (%)	13 [62]	0 (0)

BMI: Body mass index.

**Table 2:** Primary diagnoses recorded for critically ill patients with acute organ failure.

Primary pathology	n (%)
Infection	14 [67]
Hemorrhage	3 [14]
Coronary ischemia/arrhythmia/cardiogenic shock	8 [38]
Respiratory	10 [48]
Acute liver impairment	2 [9]
Acute renal impairment	3 [14]
Gastrointestinal/intra-abdominal	8 [38]

### 2.2 The bioenergetic phenotype in the first 48 h of critical illness differs from health

**Fig. 2:**
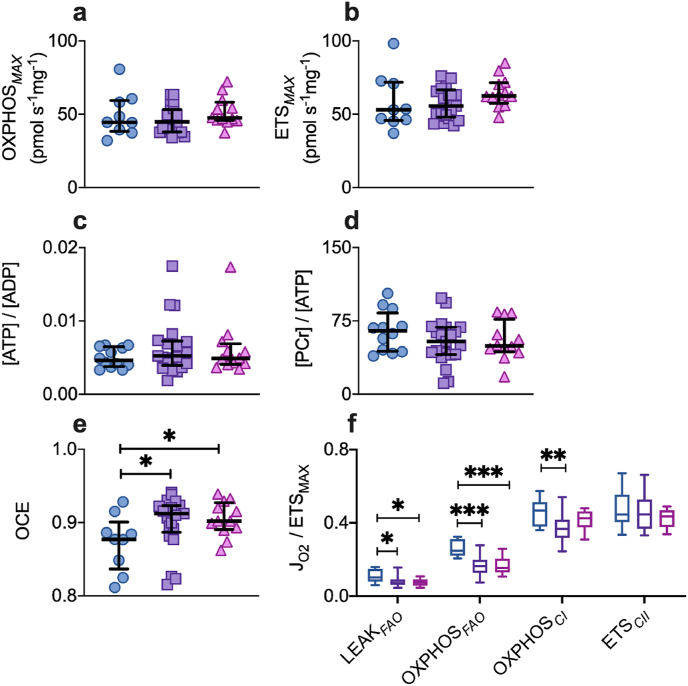
Skeletal muscle bioenergetics in critical illness. Vastus lateralis muscle from healthy reference patients (n = 9, blue circles), critically ill patients within the first 48 h of ICU admission (n = 21, purple squares) and at day 5–7 (n = 12, pink triangles). Median (IQR). a, Maximum oxidative phosphorylation capacity, stimulated by saturating convergent electron entry to the Q junction via complexes I and II and expressed per mg wet weight (OXPHOS_*MAX*_ per mass). b, Maximum capacity of the uncoupled electron transfer system (ETS_*MAX*_ per mass). c, Ratio of ATP to ADP within skeletal muscle, d, Ratio of phosphocreatine (PCr) to ATP within skeletal muscle. e, OXPHOS coupling efficiency (OCE). f, Relative capacities for respiration supported by proton leak (LEAK_*FAO*_), fatty acid oxidation (OXPHOS_*FAO*_), complex I (OXPHOS_*CI*_) and complex II (ETS_*CII*_), expressed relative to ETS_*MAX*_. Box and whisker: healthy reference group (blue); acute organ failure patients within first 48 h of admission (purple), and at day 5–7 (pink). Mann-Whitney test, two-tailed, *p < 0.05, **p < 0.01, ***p < 0.001 vs healthy reference group. (For interpretation of the references to color in this figure legend, the reader is referred to the Web version of this article.)

Oxygen flux (JO_2_) was measured in saponin-permeabilized muscle fibers using high-resolution respirometry and a substrate-uncoupler-inhibitor protocol to determine the capacity of mitochondrial respiration under different respiratory states and substrate-led pathways (Supplementary Table 3 and Supplementary Fig. 1). Individual components of the bioenergetic machinery were investigated, e.g. complex I, II and FAO-supported pathways of electron flow; and leak (LEAK), oxidative phosphorylation (OXPHOS) and uncoupled electron transfer system (ETS) respiratory states. Contrary to the prevailing doctrine of cellular bioenergetic collapse in critical illness, the maximum capacity for oxidative phosphorylation (OXPHOS_*MAX*_ per mg wet weight), under conditions of saturating convergent electron flow at the Q junction, did not differ between patients with acute organ failure and the reference group (Fig. 2a). There was also no difference between the groups in terms of maximum capacity of the electron transfer system when uncoupled from ADP phosphorylation (ETS_*MAX*_ per mg, Fig. 2b). In line with these findings, skeletal muscle ratios of [ATP]/[ADP] and [PCr]/[ATP] were also preserved in critical illness (Fig. 2c and d). However, specific alterations to the mitochondrial oxidative phosphorylation machinery were evident within 48 h of developing organ failure. Coupling efficiency was higher in the critically ill cohort at 48 h compared to the reference group (ratio 0.91 v 0.88, p = 0.045, Fig. 2e). This reflected a relative reduction in LEAK respiration in early critical illness, which was 30% lower in critically ill patients than the reference group (p = 0.02, Fig. 2f). The relative capacity for complex I-supported respiration (OXPHOS_*CI*_/ETS_*MAX*_) was also 21% lower in the critically ill cohort at 48 h compared to the reference group (p = 0.009), whilst FAO-supported respiration (OXPHOS_*FAO*_/ETS_*MAX*_) was 34% lower in the critically ill cohort than in the reference group (p < 0.001, Fig. 2f).

### 2.3 Specific and dynamic bioenergetic modifications associated with resilience

**Fig. 3:**
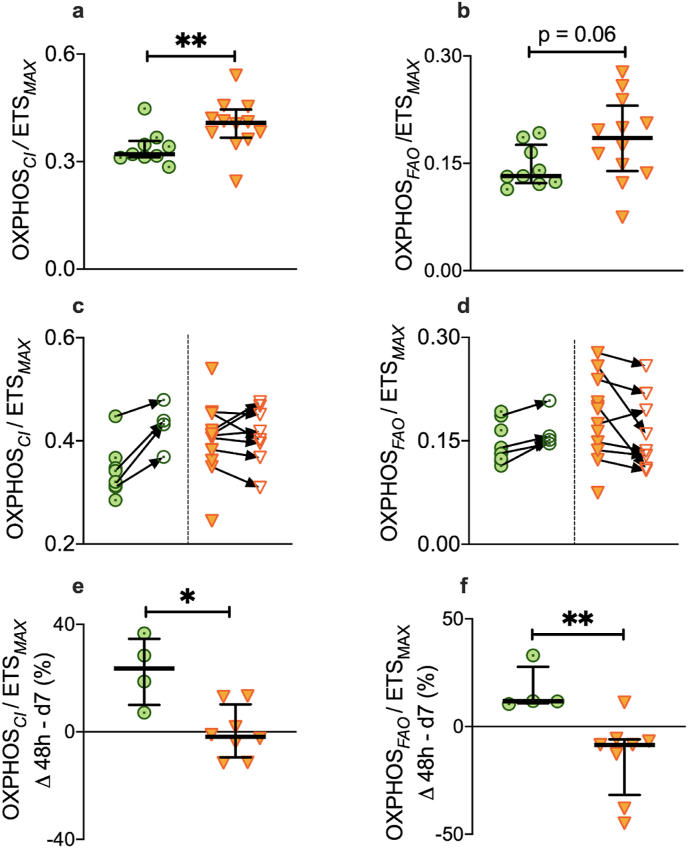
Bioenergetic divergence in survivors and non-survivors of critical illness Vastus lateralis muscle from survivors (n = 9, filled green target symbol) and non-survivors (n = 12, filled orange triangles) at < 48 h of ICU admission). Biopsy repeated at Day 5–7 in survivors (n = 4, open green target symbols) and non-survivors (n = 8, open orange triangles). Median (IQR). a, Relative capacity for respiration supported by complex I, expressed relative to ETS_*MAX*_, at < 48 h, b, Relative capacity for respiration supported by FAO, expressed relative to ETS_*MAX*_, at < 48 h c, Relative complex I respiratory capacity measured at both <48 h and Day 5–7. d, Relative FAO respiratory capacity measured at both <48 h and Day 5–7. e, Percentage change in relative complex I respiratory capacity from <48 h to Day 5–7. f, Percentage change in relative FAO respiratory capacity from <48 h to Day 5–7. Mann-Whitney test, two-tailed. *p < 0.05, **p < 0.01. (For interpretation of the references to color in this figure legend, the reader is referred to the Web version of this article.)

In the first 48 h of critical illness, relative complex I capacity was 27% lower in eventual survivors compared to non-survivors (p = 0.01, Fig. 3a). There was a trend toward a 40% lower relative FAO capacity in eventual survivors compared to patients who died (p = 0.06, Fig. 3b). The percentage change in these specific respiratory capacities, from 48 h to day 5–7, also differed significantly between survivors and non-survivors (Fig. 3). All survivors underwent an increase in their capacity for complex I-mediated respiration (median change +24%; IQR 10–35) in contrast to the non-survivors, in whom the diminished complex I capacity persisted (median change −1.8%; IQR -9.5-10.3, p = 0.016, Fig. 3c, e). Capacity for FAO-supported respiration underwent an increase in survivors by day 5–7 (median change +12%; IQR 11–28), in contrast to non-survivors who underwent a median change of −8.5%; IQR -32 to −6, p = 0.008, Fig. 3d,f).

### 2.4 Lipid overload is a corollary of bioenergetic remodeling in acute organ failure

**Fig. 4:**
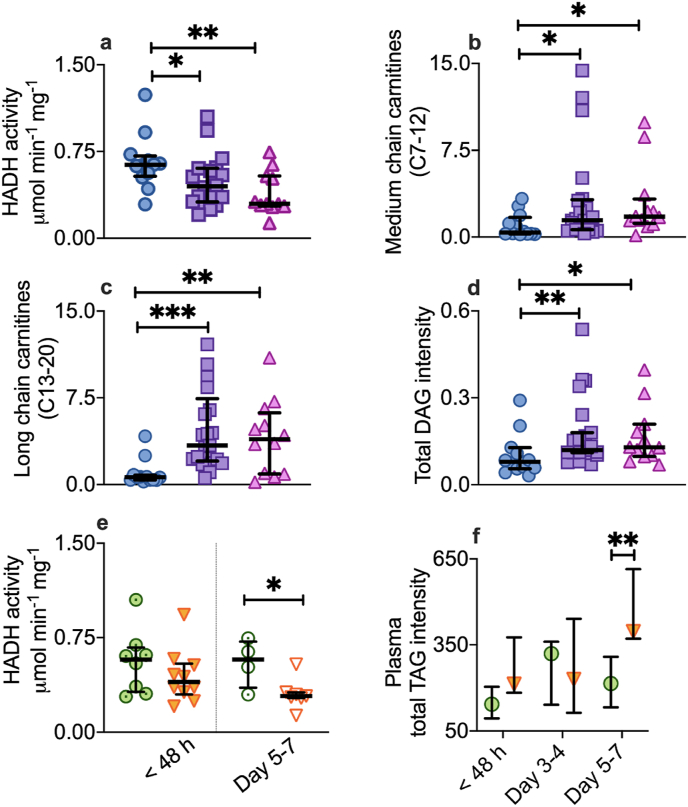
Skeletal muscle and systemic lipid signals in critical illness. a-e, Vastus lateralis muscle from healthy reference patients (n = 9, blue circles), critically ill patients within the first 48 h of ICU admission (n = 21, purple squares) and at Day 5–7 (n = 12, pink triangles). Metabolite values expressed relative to the average of the healthy reference group. a, Mass-specific activity of 3-hydroxyacyl-CoA dehydrogenase (HADH) in skeletal muscle. b, Intramuscular ratio of medium chain (carbon chain length 7–12) to total carnitines. c, Intramuscular ratio of long chain (carbon chain length 13–20) to total carnitines. d, Intramuscular intensity of diacylglycerols (DAG). e, Mass-specific HADH activity at < 48 h in survivors (n = 9, green filled target symbol) and non-survivors (n = 12, orange filled triangles), and at Day 5–7 (survivors, white target symbols, n = 4, non-survivors, white triangles, n = 12). f, Total triacylglycerol (TAG) in venous plasma, in survivors (green circles; n = 9 at < 48 h, 7 at Day 3–4, 6 at Day 5–7) and non-survivors (orange triangles; n = 11 at < 48 h, 8 at Day 3–4, 8 at Day 5–7). Median (IQR). Mann-Whitney test, two-tailed. *p < 0.05, **p < 0.01, ***p < 0.001, ****p < 0.0001. (For interpretation of the references to color in this figure legend, the reader is referred to the Web version of this article.)

Concomitant muscle and plasma lipidomic analyses provided insight into the metabolic consequences of the altered mitochondrial substrate utilization in critical illness. Targeted metabolomic analysis was carried out on muscle homogenates and open-profile lipidomics on muscle and plasma using ultra-high performance liquid chromatography mass spectrometry and relevant muscle enzyme activities were assessed using spectrophotometric assays. In skeletal muscle, the reduced mitochondrial capacity for respiration supported by FAO in the first 48 h of critical illness was consistent with diminished activity of the FAO enzyme, 3-hydroxyacyl-CoA dehydrogenase (HADH, Fig. 4a), which was 29% lower in the critically ill cohort compared to the reference group (p = 0.03). It was also associated with relative accumulation of medium (carbon (C) 7–12) and long (C13-20) chain acyl-carnitines compared to the reference group (p = 0.02 and < 0.001 respectively, Fig. 4b and c). Skeletal muscle lipidomic analysis also revealed accumulation of total diacylglycerol (DAG, C30-46), levels of which were markedly greater in the critically ill cohort than the reference group (p = 0.007, Fig. 4d). This picture of skeletal muscle lipid backlog fits with the observed diminished mitochondrial capacity for FAO. In support of the earlier finding that failure to recover FAO metabolism at day 5–7 was a hallmark of poor outcome, by day 5–7 HADH activity was 50% lower in non-survivors compared to survivors (p = 0.048, Fig. 4e). The progressive imbalance between lipid supply and demand also existed at the systemic level, as plasma lipidomic analysis revealed a markedly greater intensity of venous plasma total triacylglycerol (TAG, C37-62) in non-survivors compared to survivors at day 5–7 (p = 0.003, Fig. 4f).

### 2.5 Alterations to intermediary metabolism are consistent with a cellular response to hypoxia

**Fig. 5:**
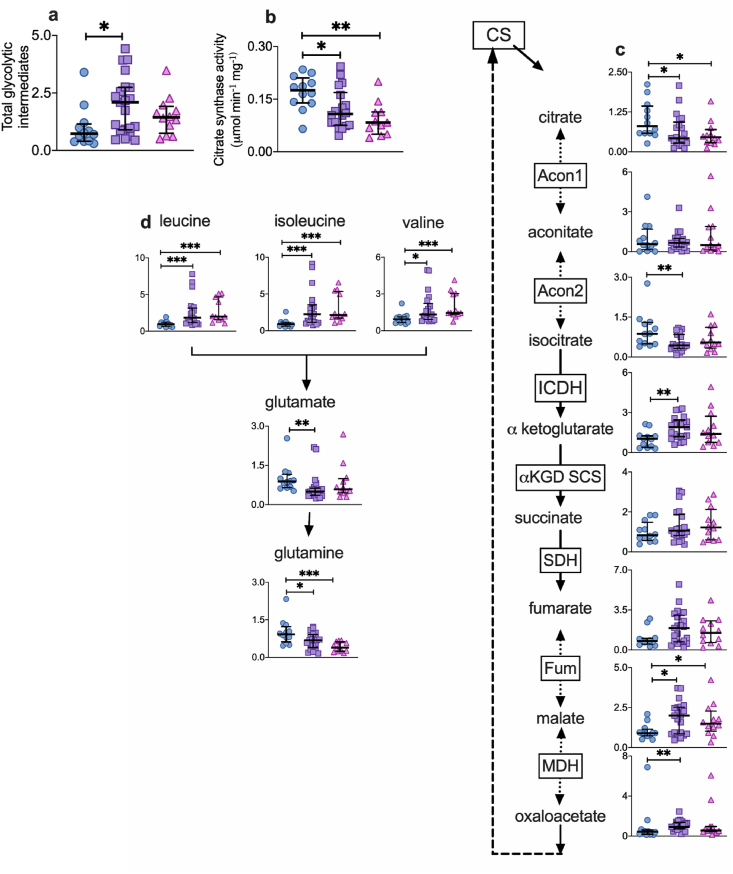
**Metabolic pathway alterations in skeletal muscle during critical illness.** Vastus lateralis muscle from healthy reference patients (n = 9, yellow circles), critically ill patients within the first 48 h of ICU admission (n = 21, blue squares) and at Day 5–7 (n = 12, pink triangles). Metabolites measured using ultra-high performance liquid chromatography mass spectrometry, and values expressed relative to the average of the healthy reference group. Median (IQR). a, Total glycolytic intermediates. b, Citrate synthase enzyme activity, measured spectrophotometrically, expressed relative to protein concentration, c, Intermediates of the tricarboxylic acid pathway, d, Selected amino acids. Mann-Whitney test, two-tailed. *p < 0.05, **p < 0.01, ***p < 0.001, ****p < 0.0001 vs the reference group. Enzyme key. CS: citrate synthase; Acon1: aconitase 1; Acon2: aconitase 2; ICDH: isocitrate dehydrogenase; ⍺-KGD SCS: ⍺-ketoglutarate dehydrogenase; SDH: succinate dehydrogenase; Fum: fumarase; MDH: malate dehydrogenase. (For interpretation of the references to color in this figure legend, the reader is referred to the Web version of this article.)

Critically ill patients demonstrated hallmarks of a coordinated metabolic skeletal muscle response to hypoxia, with evidence for upregulation of glycolysis and shunting of pyruvate away from the TCA cycle (and oxidative phosphorylation) toward lactate production, as demonstrated by elevated levels of total glycolytic intermediates (3-fold greater in the critically ill cohort compared to the reference group, p = 0.01, Fig. 5a), particularly pyruvate, which occurred in parallel with reduced levels of early (6-carbon) TCA cycle intermediates, and reduced citrate synthase activity (Fig. 5b and c). In critical illness, elevation in the latter (5- and 4-carbon) metabolites of the TCA cycle, despite the depletion in the early intermediates (Fig. 5c), suggests the existence of an alternative pathway for replenishing the cycle, via α-ketoglutarate and increased malate-aspartate shuttle activity. Glutamine, and its precursor, glutamate, were indeed lower in early organ failure (Fig. 5d), consistent with a diversion of amino acids to replenish the TCA cycle.

### 2.6 Redox status is associated with survival in organ failure

**Fig. 6:**
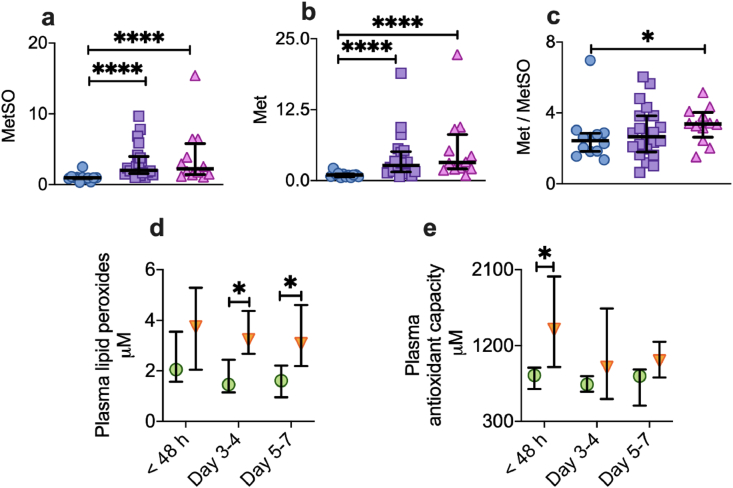
Redox modifications during critical illness a-c, Vastus lateralis muscle from healthy reference patients (n = 9, blue circles), critically ill patients within the first 48 h of ICU admission (n = 21, purple squares) and at Day 5–7 (n = 12, pink triangles). Metabolites expressed relative to the average of the healthy reference group. Median (IQR). Mann-Whitney test, two tailed. *p < 0.05, **p < 0.01, ***p < 0.001, ****p < 0.0001 vs healthy reference group. a, Intramuscular levels of the oxidation product of methionine, methionine sulfoxide (MetSO), b, Intramuscular reduced methionine (Met) and c, Ratio of reduced Met to its oxidation product, MetSO, in skeletal muscle. d-e, Venous plasma redox markers in survivors (green circles; n = 9 at < 48 h, 7 at Day 3–4, 6 at Day 5–7) vs non-survivors (orange triangles; n = 11 at < 48 h, 8 at Day 3–4, 8 at Day 5–7). Median (IQR). Mann-Whitney, two tailed. *p < 0.05. d, Plasma lipid peroxides and e, total antioxidant capacity. (For interpretation of the references to color in this figure legend, the reader is referred to the Web version of this article.)

A complementary array of assays was used to assess different components of redox status in muscle, plasma and erythrocytes. We found higher intramuscular levels of the oxidized component of the redox couple methionine/methionine sulfoxide in critically ill patients compared to the reference group (Fig. 6a). However, critically ill patients had even higher levels of the reduced form compared to the reference group (Fig. 6b and c). Instead of a simple shift toward uncompensated oxidative stress in critical illness, suggested by previous single biomarker approaches, these findings indicate an enhanced adaptive redox drive within skeletal muscle, with upregulation of cellular antioxidant capacity in the face of an increased oxidative burden. The same process was demonstrated at the systemic level. Non-survivors showed greater levels of whole-body oxidative stress than survivors, in the form of lipid oxidation products in venous plasma (Fig. 6d), but, also greater plasma reducing capacity at <48 h (Fig. 6e). Together, these data suggest that an active upregulation of reducing power, in both tissue (muscle) and systemic (plasma) compartments, occurs in response to life-threatening stress and oxidant injury, with non-survivors succumbing despite even greater enhancement of reducing capacity. In another compartment, venous erythrocytes, there was also a non-significant trend towards higher reducing capacity in non-survivors in the first 48 h of critical illness, according to the relative concentrations of reduced/oxidized glutathione (Supplementary Fig. 2). In addition, changes in the concentration of the powerful antioxidant glutathione persulfide (GSSH) were observed with divergent trajectories between survivors and non-survivors, although this did not meet criteria for statistical significance.

### 2.7 Nitric oxide production/metabolism related to survival but not bioenergetic alterations

**Fig. 7:**
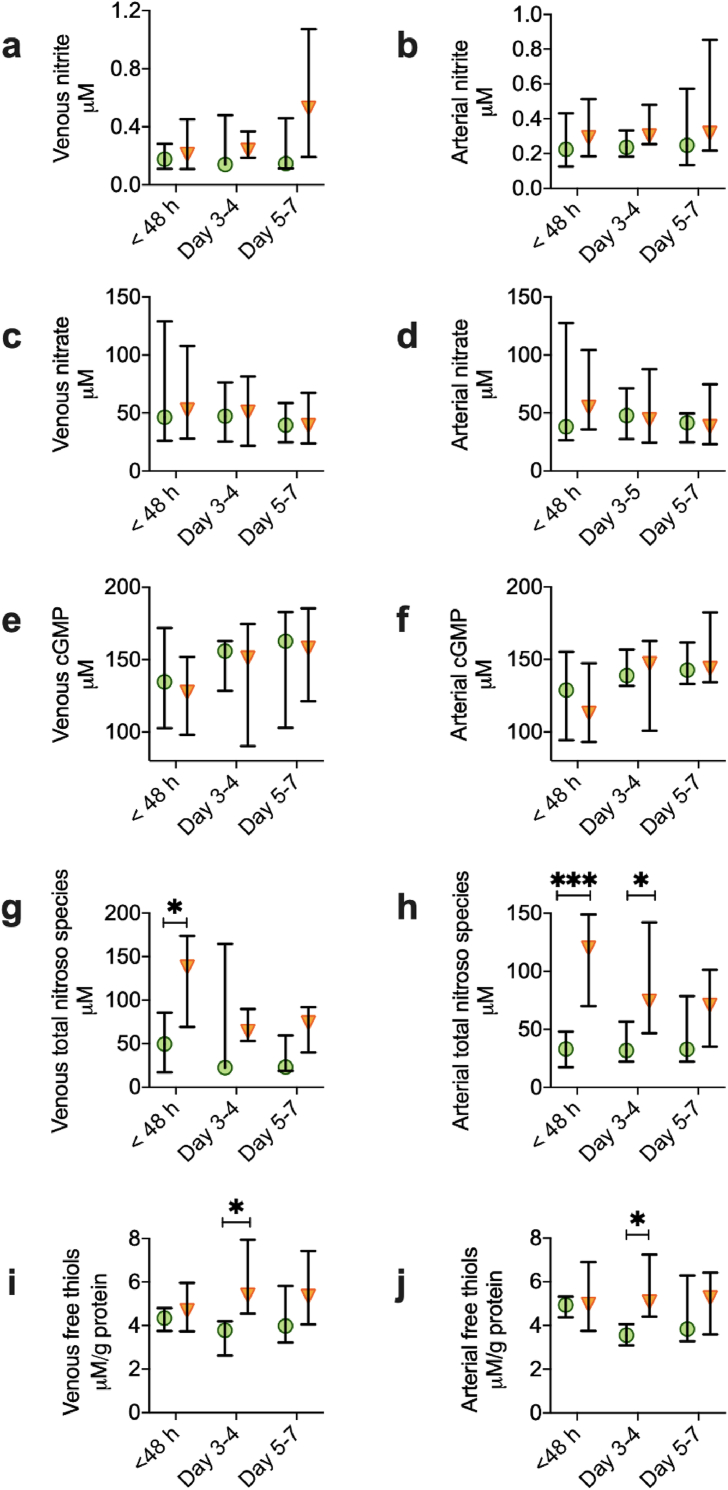
**Nitric oxide availability in survivors and non-survivors of critical illness** Circulating concentrations of markers of NO production and metabolism in venous plasma from critically ill survivors (green circles; n = 9 at < 48 h, 7 at Day 3–4, 6 at Day 5–7) vs non-survivors (orange triangles; n = 11 at < 48 h, 8 at Day 3–4, 8 at Day 5–7), and in arterial plasma from critically ill survivors (green circles; n = 9 at < 48 h, 7 at Day 3–4, 4 at Day 5–7) vs non-survivors (orange triangles; n = 12 at < 48 h, 9 at Day 3–4, 8 at Day 5–7). a, venous nitrite, b, arterial nitrite, c, venous nitrate, d, arterial nitrate, e, venous cGMP, f, arterial cGMP, g, venous total nitroso species, h, arterial total nitroso species, i, venous total free thiols, j, arterial total free thiols (both corrected for protein). Median (IQR). Mann-Whitney, two tailed. *p < 0.05, **p < 0.01. ***p < 0.001.

Although there was no difference in steady-state concentration between critically ill survivors and non-survivors at any timepoint in terms of venous plasma nitrite, nitrate or cGMP, total nitroso species in non-survivors were 178% higher than those in survivors at <48 h (p = 0.016, Fig. 7). The same pattern was seen in arterial plasma, with no differences in nitrite, nitrate or cGMP, suggesting no changes in NO availability (cGMP) or total body NO production (nitrate). By contrast, arterial plasma levels of total nitroso species in non-survivors were 260% higher than those in survivors at < 48 h (p < 0.001) and 133% higher than non-survivors at day 3–4 (p = 0.04, Fig. 7). These differences attest to marked differences in vascular inflammatory status and associated nitrosative stress between the two groups. At day 3–4, serum total free thiols (normalized per g protein) were also 43% higher in venous plasma of non-survivors compared to survivors (p = 0.01), and 42% higher in the arterial plasma of non-survivors compared to survivors (p = 0.04, Fig. 7). There were no significant associations between any of these plasma markers of NO production and metabolism and the redox ratios measured in skeletal muscle.

### 2.8 Relationship between critical illness phenotype and markers of inflammation

There was no difference between survivors and non-survivors in terms of the traditional clinical markers of inflammation, such as serum C-reactive protein (CRP) or white cell count (WCC), at any of the three timepoints, as shown in Supplementary Data Fig. 3.

There was also no relationship between levels of these clinical inflammatory markers and any of the plasma indicators of redox status and NO metabolism, measured in critically ill patients. Thus, the differences between survivors and non-survivors appear unlikely to represent differences in the magnitude of systemic inflammation alone.

## 3 Discussion

This work supports the concept that critical illness alters the machinery governing cellular bioenergetic capacity and substrate metabolism and highlights specific modifications that are associated with human resilience during life-threatening stress. Despite the moderate number of patients investigated and the overall heterogeneity of the critically ill cohort, there was a coherent and internally consistent profile of modifications associated with multiple-organ failure. This involved cellular respiratory capacity, intermediary metabolism and redox architecture, which differed from health; and although none of the critically ill patients were hypoxemic, the profile of alterations observed bore many similarities to the cellular response to hypoxia. Specific aspects of the critical illness phenotype appeared to confer resilience, with greater initial divergence from normality in complex I and FAO-supported respiratory capacity associated with survival. These particular mitochondrial modifications may promote bioenergetic efficiency with respect to oxygen consumption, and thus contribute to preservation of muscle energetics despite the diminished capacity for complex I mediated respiration. Increased coupling efficiency reflects a reduction in mitochondrial proton leak relative to OXPHOS and means a greater proportion of the substrate and oxygen consumption that drive mitochondrial electron transport are channelled towards ATP production [20]. The substrate switch away from FAO will also increase bioenergetic efficiency, as respiration supported by FAO produces fewer ATP molecules per mole of oxygen consumed, compared with carbohydrate oxidation [21]. This pattern of substrate reprogramming is characteristic of a metabolic response to cellular hypoxia; it has been described in the heart of the human fetus developing under relatively low oxygen tensions in utero [22] and can be induced in adult human cardiac muscle in response to cellular oxygen limitation [23]. Himalayan Sherpas have a lower skeletal muscle capacity for FAO-supported respiration and greater coupling efficiency, compared to lowlanders; and this has been proposed to underpin their augmented cellular energetics and superior performance under conditions of hypobaric hypoxia at high altitude [12].

The pattern of bioenergetic and metabolic modifications in critical illness suggests a potential role for hypoxia inducible factor-1α (HIF-1α) in coordinating this response. Intracellular levels of HIF-1α are known to increase in response to reduced cellular oxygen availability, and to activate the transcription of genes involved in the adaptive response to hypoxia [24]. In cell models of hypoxia, HIF has been shown to mediate responses which promote survival under hypoxic conditions and which bear a striking similarity to the critical illness skeletal muscle phenotype described in this work, including: increased efficiency of oxidative phosphorylation [25]; reduced FAO [26]; increased transcription of glycolytic enzymes and lactate dehydrogenase (promoting conversion of pyruvate to lactate) as well as pyruvate dehydrogenase kinase 1 (shunting pyruvate away from mitochondria) [27,28]. Although none of the critically ill patients were hypoxemic, their tissue oxygen partial pressure was not measured. In multiple-organ failure, cellular oxygen supply may be limited, even when cardiac output and convective oxygen transport are resuscitated, due to microcirculatory disturbance and inflammation [29]. Intramuscular succinate levels, also a potential marker of cellular hypoxia during tissue ischemia [30], were similar in critically ill patients and the reference group. However, elevated skeletal muscle concentrations of HIF-1α have been described in a cohort of critically ill patients, similar to that in the current study, even in the presence of normal arterial oxygen indices [16]. An alternative pathway for HIF-stabilisation in this context could be mitochondrially-generated ROS, which have been implicated in the induction of the HIF transcriptional response [31], whilst ROS stabilise the HIF α-subunit even under normoxic conditions [32]. This mechanism would be consistent with the elevated production of cellular and systemic oxidants in critical illness, demonstrated in the current study.

Demonstration of reduced complex I respiratory capacity in the critically ill cohort was consistent with the reduced expression and activity of complex I in frozen muscle previously reported in critical illness [15,33], with lower activity previously associated with greater severity of shock [17]. Here we demonstrated for the first time that these protein changes have functional relevance and translate into altered respiratory capacity in intact mitochondria. Instead of reflecting mitochondrial dysfunction, however, early downregulation of complex I in critical illness appears to be protective, with eventual survivors demonstrating significantly lower complex I capacity than non-survivors within the first 48 h. The reduced capacity for complex I-supported respiration in critical illness also fits in with the concept of a hypoxia-induced adaptation response [13]. Downregulation of complex I in skeletal muscle of lowlanders acclimatizing to hypobaric hypoxia has been described as an adaptive response to protect the cell against enhanced ROS production [34]. In human placental tissue, the mechanism underlying hypoxic downregulation of complex I has been shown to involve the HIF-responsive microRNA-210, which represses iron-sulfur protein assembly of complex I under very low oxygen conditions [13]. However, the effects of HIF on the electron transport chain previously described are not specific to complex I, but encompass the downregulation of other respiratory complexes. In this work, however, the diminished complex I capacity occurred in isolation, whilst maximum respiratory capacity was preserved, with no evidence for a compensatory increase in contribution from complex II. The association of free-moving complexes into supercomplexes may be relevant to this phenomenon, as both the relative abundance and specific composition of supercomplexes have previously been shown to alter in response to changes in cellular metabolic demand [35,36].

An alternative explanation for isolated downregulation of complex I demonstrated in this work, is via active/deactive transition of complex I, whereby the catalytically active form of complex I undergoes deactivation to a dormant state, when the activity of the respiratory chain is limited, such as during hypoxia [37]. The mechanism behind this transition is not completely understood, but involves conformational changes in mitochondrially encoded subunits, exposing a critical cysteine residue on subunit ND3, which renders the deactive form susceptible to nitrosation and thiol oxidation [37]. Complex I is a major site for the generation of ROS, particularly superoxide [38]. Complex I can produce ROS by both forward and reverse electron flow, with the latter responsible for the oxidative burst upon reoxygenation [36]. Hypoxia-induced complex I deactivation is thought to drive ROS production at complex III [38], and via reversal of TCA cycle dehydrogenases [39], resulting in stabilisation of HIF-1α [40]. In contrast, when complex I is in the deactive form, superoxide production from reverse electron flow is suppressed, and this is thought to protect mitochondria from excessive ROS production upon reoxygenation [37]. Taken together, the data presented here may be consistent with acute hypoxia in cells of critically ill patients triggering a deactive transition in complex I, resulting in ROS release and stabilisation of HIF, thereby generating the metabolic phenotype of downregulated FAO and increased glycolysis.

The temporal nature of the respiratory modifications was crucial, with the early deviations in complex I and FAO capacities tending to recover by day 5–7 in survivors, whilst the downregulation tended to persist, or progress, in non-survivors at the later timepoint. The divergent trend between survivors and non-survivors in the dynamics of these alterations reveals the importance of the timing of engagement and release of this “brake” for patient survival. The fact that non-survivors tended not to decrease complex I and FAO-dependent respiration as much as survivors during the first 48 h of critical illness may indicate faster and more effective hypoxic acclimation by survivors; who can then return to normal more rapidly. Non-survivors, on the other hand, appear to not adapt to cellular hypoxia so effectively, such that several days later, hypoxic acclimation is still ongoing. Inferior hypoxic acclimation in the non-survivors could contribute to greater energy imbalance and cellular dysfunction. This may be further compounded by the cost of the diminished or delayed metabolic/bioenergetic modifications being prolonged over a longer time period. For example, glycolysis was upregulated in critically ill muscle, and may have contributed to the preservation of intramuscular energetics under oxygen limited conditions. Anabolic pathways, such as the conversion of lactate to glucose via the Cori cycle, may require the investment of ATP in the liver [41]. Increased glucogenesis may also increase the metabolism of glutamate, glutamine and other glucogenic amino acids to replenish TCA cycle intermediates via anaplerosis. Their depletion may be part of the mechanism of rapid muscle wasting in critical illness [42], as they play an important role in inhibiting protein degradation and stimulating protein synthesis [43]. Rapidly dividing cells such as immune cells and the cells of the gastrointestinal tract also have a high demand for glutamine as a substrate [43].

Another cost of increased mitochondrial efficiency could be related to the downstream sequelae of reduced mitochondrial preference for fatty acids as a respiratory substrate, with the accompanying impairment in the skeletal muscle FAO pathway, and consequent accumulation of intramuscular medium and long-chain acylcarnitines and diacylglycerol. The concept that failure to recover cellular FAO metabolism is a hallmark of poor outcome was supported by the finding that muscle HADH activity was significantly lower in non-survivors by day 5–7, and may have contributed to progression of imbalance between lipid supply and demand at the systemic level, with markedly greater intensity of venous total TAG at the later timepoint. Ectopic (non-adipose) lipid deposition has been associated with metabolic dysfunction and cell toxicity [44]. In particular, the accumulation of muscle DAGs in the present study may have further implications for metabolic regulation through insulin signaling, as muscle DAG accumulation has been linked to induction of insulin resistance via protein kinase C [45,46]. Acute onset insulin resistance is well documented in critical illness and has been attributed to activation of the hypothalamic-pituitary-adrenal axis [47]. The present study suggests an additional mechanism, although protein kinase C and insulin resistance were not measured. Reduced skeletal muscle FAO in organ failure was accompanied by accumulation of muscle branched-chain amino acids (BCAAs: leucine, isoleucine and valine, Fig. 5d). Unlike other amino acids, BCAAs are primarily metabolized in muscle, hence this finding implies reduced muscle consumption. The reduced activity of HADH may contribute to impaired BCAA oxidation, which has also been associated with the development of insulin resistance [[48], [49], [50]]. High plasma TAGs can lead to ectopic lipid deposition in skeletal muscle, including the accumulation of DAGs via the activity of lipoprotein lipase [51,52]. Elevation of circulating TAGs is known to contribute to metabolic syndrome [53], and represents a risk factor for type 2 diabetes and cardiovascular disease [54]. In critically ill patients, exposure to intravenous lipid infusions has been shown to have a detrimental effect of enhancing inflammatory responses [55]. Indeed, the higher circulating levels of nitroso species in plasma of non-survivors compared to survivors, *vis-a-vis* similar concentrations of nitrite, nitrate and cGMP (indicative of no changes in either NO production or availability) are indicative of a greater level of oxidative stress with enhanced production of superoxide (and consecutive formation of peroxynitrite) due to vascular inflammation in non-survivors; whether inflammation is restricted to the endothelium or extends to also include smooth muscle and/or the adventitial compartment remains unknown. Endothelial dysfunction is a hallmark of vascular inflammation, associated with enhanced platelet and leukocyte activation, and has recently been advocated to be targeted in order to reduce organ failure in critical illness [56]. Taken together, these data support the concept that the initial survival advantage gained by augmenting bioenergetic efficiency may ultimately come at the cost of disruption of metabolic homeostasis and downstream signaling, which if sustained, is incompatible with life.

Organ failure and risk of death have previously been associated with increased by-products of oxidative stress [57] and correspondingly, we found higher intramuscular levels of the oxidized components of the redox couple, methionine/methionine sulfoxide. However, instead of a simple shift towards uncompensated oxidative stress in organ failure, as suggested by previous studies using single biomarker approaches, this work demonstrated the presence of a compensatory increase in reductive drive within skeletal muscle in critical illness, suggesting upregulation of cellular antioxidant capacity in the face of an increased oxidative burden. The same process was demonstrated at the whole-body level, with non-survival associated with greater oxidative injury (in the form of increased lipid oxidation products) alongside increased total reducing power in plasma. Together, these data suggest that an active upregulation of reducing power in both tissue (muscle) and systemic (plasma) compartments occurs in response to life-threatening stress and oxidant injury, with non-survivors succumbing despite even greater enhancement of reducing capacity.

This finding of exaggerated reducing power in non-survivors was mirrored by corresponding changes in plasma total free thiol levels (which largely reflect the concentration of the single free cysteine thiol of serum albumin) and was consistent with that of a previous study [58]. In contrast, the redox status alterations in a third compartment, venous erythrocytes, were indicative of progressive oxidative stress in non-survivors (Supplementary Fig. 2), which may be a result of systems-wide coupling of redox processes for whole-body balance (thus, the price for enhanced reducing capacity in one compartment is increased oxidative stress elsewhere) [14]. Importantly, as with temporal divergence between survivors and non-survivors in bioenergetic modifications, erythrocyte glutathione redox status and concentrations of the powerful antioxidant, glutathione persulfide (GSSH) evolved into opposite directions in survivors and non-survivors, with considerable inter-individual heterogeneity (Supplementary Fig. 2). The complexity of these redox changes only becomes apparent from a high-resolution analysis of multiple redox components across different compartments, demonstrating the limitation of assessing ‘redox status’ by using a small number of biomarkers in isolation.

### 3.1 Limitations

The observed differences between the critically ill phenotype and reference subjects, and between the survivor phenotype and non-survivors, must be interpreted with caution. The description of these phenotypes is limited by the fact that this is a single study, in a small group of highly heterogenous patients with a high attrition rate, which increases the risk of type II error. Despite this, statistically significant differences between survivors and non-survivors were detected in several variables; even at the third timepoint, when subject numbers had decreased, implying that for these variables at least, power was adequate. There was a high potential for confounding factors to influence outcomes: and although no relationship was demonstrated between, for example, bioenergetic changes and nutritional intake or medications, it is impossible to rule out that a combination of differences between the reference cohort and the organ failure cohort, other than acute organ failure, may have contributed to differences between the groups. The risk of type I error was increased by the multiple statistical tests and correlations tested within this dataset. However, in this exploratory study, where the aim was to discover potentially important differences, in a highly unstable clinical cohort, the priority was to optimise sensitivity to detect potential signals in an exploratory fashion, which can then be further evaluated in dedicated future studies. By using a combination of approaches to describe a complex phenotype, this data set also demonstrated considerable internal consistency with lower mitochondrial capacity for FAO-supported respiration accompanied by lower FAO enzyme activity and evidence for incomplete breakdown of longer-chain carnitines in muscle, as well as accumulation of TAG in plasma.

One important limitation of mitochondrial analysis using permeabilised muscle fibers is the loss of potentially relevant interactions between respiratory complex activity and TCA cycle intermediates, due to the washout of metabolites and cations. For example, our metabolomic analysis demonstrated an accumulation of the TCA cycle intermediate, oxaloacetate, in the skeletal muscle of critically ill patients. Oxaloacetate is known to be a high affinity inhibitor of complex II [59], however in the ex vivo assessment of respiration, critically ill patients did not differ from the reference group in terms of complex II capacity (Fig. 1f, Table 3).

**Table 3:** Mass-specific respiration rates in the critically ill and reference cohorts.

J_O2_ per mass (pmol/s/mg wet weight) median (IQR)	Reference group (n = 9)	Critically ill patients < 48 h (n = 21)	Critically ill patients day 5–7 (n = 12)
LEAK_*FAO*_	5.49 (4.82–6.75)	4.55 (3.17–5.51) *	4.54 (3.62–6.02)
OXPHOS_*FAO*_	14.7 (10.5–17.1)	9.96 (7.16–10.8) **	9.25 (7.20–13.4) *
OXPHOS_*CI*_	26.2 (19.6–31.3)	20.5 (17.3–23.5)	26.8 (22.0–30.8)^##^
OXPHOS_*MAX*_	44.6 (38.4–59.4)	44.8 (38.0–53.2)	47.5 (45.9–58.2)
ETS_*MAX*_	53.0 (45.8–71.8)	55.59 (48.0–66.6)	62.6 (57.5–71.5)^##^
ETS_*CII*_	24.3 (21.0–32.9)	24.5 (22.5–27.8)	25.5 (22.2–32.3)

Respiratory capacities generated by sequential addition of substrates, uncouplers and inhibitors according to previously described SUIT protocol. JO_2_: oxygen consumption rate at steady state; LEAK_*FAO*_: capacity for respiration supported by proton leak, in the presence of malate and octanoyl carnitine and absence of ADP; OXPHOS_*FAO*_: capacity for oxidative phosphorylation supported by FAO, following addition of saturating ADP; OXPHOS_*CI*_: capacity for oxidative phosphorylation supported by complex I; following addition of saturating pyruvate and glutamate; OXPHOS_*MAX*_: maximum capacity for oxidative phosphorylation supported by complex I and complex II, following addition of succinate; ETS_*MAX*_: maximum capacity of the electron transport system uncoupled from oxidative phosphorylation, following titration of uncoupler; ETS_*CII*_; capacity of uncoupled electron transport system supported by complex II alone, following inhibition of complex I by rotenone. *p < 0.05, **p < 0.01 vs reference group; ^#^p < 0.05, ^##^p < 0.01 vs critically ill patients within 48 h.

Although the comprehensive phenotype of bioenergetic, metabolic and redox modifications described here suggest a possible role for hypoxic adaptation and metabolic regulation in the cellular response to critical illness, such a mechanism cannot be demonstrated using these data. Further elucidating this potential mechanism will require assessment of other markers of HIF activation and its downstream targets. Although these pathways have not yet been explored in critically ill patients, they are thought to play a role in cellular adaptation to stress in humans in related contexts, such as acclimatization to hypobaric hypoxia, fetal adaptation to the intrauterine environment and substrate switching in chronic heart failure [60].

## 4 Conclusion

The failure to consider the potential relevance of intracellular adaptive modifications to stress in the context of critical illness may be preventing important therapeutic advances. From these data, evidence emerges for the existence of a specific end-organ cellular phenotype, expressed in real-world human critical illness, that is associated with stress resilience. Survival may rely on both the cellular ability to launch an appropriate adaptive response at the onset of a critical insult as well as the ability to switch it off and/or initiate appropriate counter-regulation in a timely manner, to allow metabolic recovery and minimize harmful consequences of bioenergetic and redox compensation. To this end, it will be important to capture the time-course of events at multiple levels. Future studies should seek to confirm the current findings in larger numbers of patients, embracing the complexity of bioenergetic and redox pathways to search for solutions to human survival and long-term health post critical illness.

## 5 Methods

### 5.1 Study subjects

The study schedule is summarized in Fig. 1. All subjects were recruited from a tertiary referral teaching hospital in London, United Kingdom (the Royal Free Hospital), between March 2016 and 2017. A cohort of patients with organ failure were recruited from a mixed medical and surgical ICU. Inclusion criteria were adults (aged >18 yr) with acute onset, severe physiological impairment from any primary cause, requiring at least two forms of organ support (e.g. mechanical ventilation, vasopressor or inotropic support, renal replacement therapy) admitted to the ICU for <24 h. Patients with severe coagulopathy, primary neuromuscular pathology, disseminated cancer, therapeutic immunosuppression or diagnosed mitochondrial disorder were excluded. The primary diagnoses of the organ failure cohort are summarized in Table 2. The cohort of healthy reference subjects was recruited from the orthopedic department, and comprised adults undergoing elective hip replacement. Skeletal muscle was sampled from these patients intraoperatively under general anesthesia. In brief, the reference patients had undergone recent induction of anesthesia with intravenous opiates (usually fentanyl) and propofol, muscle relaxation using atracurium or rocuronium, and placement of an endotracheal tube for mechanical ventilation and maintenance of anesthesia with volatile agents such as isoflurane, sevoflurane or desflurane. An audit carried out at the Royal Free Hospital, along with 28 other hospitals in England, during the time of the study, demonstrated the administration of a consistent fractional concentration of oxygen of 0.5 throughout surgery, which corresponded to a measured mean partial pressure of oxygen (PaO2) of 25 kPa in patients with an arterial catheter [61]. All elective surgical patients had fasted for at least 6 h prior to induction of general anesthetic. The demographics and baseline clinical characteristics of the critically ill cohort (at <48 h from ICU admission) and healthy reference group are summarized in Table 1. In the critically ill cohort, skeletal muscle and blood were sampled within 48 h of admission to the ICU (n = 21), and repeated, when possible at day 5–7 (n = 12 for muscle, due to the death of 4 and withdrawal of 5 subjects). An additional blood sample was taken at day 3–4 (n = 16). The clinical characteristics, including relevant medication and nutritional intake, of the organ failure cohort at all three timepoints are summarized in Supplementary Table 1. Of the organ failure cohort tested within 48 h of admission to ICU, 12 died prior to hospital discharge. For the patients tested day 5–7 (n = 12), 8 died prior to hospital discharge. The clinical characteristics (measured at <48 h) of the survivors and non-survivors are summarized in Supplementary Table 2.

### 5.2 Muscle sample collection and preparation

Biopsies of the vastus lateralis muscle were taken from the mid-thigh using Tilley-Henckel forceps under local anesthesia (1% lidocaine) of the skin and superficial muscle fascia. A 5 mm incision was made and 100 mg wet-weight tissue was collected. The sample was divided, with 50 mg allocated for immediate respirometric analysis and the remainder snap frozen in liquid nitrogen and stored at −80 °C until later analysis. The muscle sample for respirometry was immediately placed in ice-cold biopsy preservation medium (BIOPS): [CaK_2_EGTA (2.77 mM), K_2_EGTA (7.23 mM), MgCl_2_.6H_2_O (6.56 mM), taurine (20 mM), PCr (15 mM), imidazole (20 mM), DTT (0.5 mM), MES (50 mM) and Na_2_ATP (5.77 mM) at pH 7.10], which was filtered and stored at −40 °C until use to prevent bacterial growth.

### 5.3 Blood sampling and preparation

Blood was sampled from the critically ill cohort via indwelling catheters (arterial and central venous catheters), placed as part of their treatment on the ICU. Aliquots were collected with EDTA as anticoagulant and immediately subjected to centrifugation to obtain plasma (800×*g* for 15 min) and a red blood cell pellet, both of which were stored at −80 °C until later analysis.

### 5.4 High-resolution respirometry

Skeletal muscle fiber bundles were prepared from the respirometry-designated sample according to previously described methods [62]. After permeabilization of the sarcolemmal membrane using saponin (50 μg/ml, in ice cold BIOPS, rocked for 20 min at 20 rpm), fiber bundles were rinsed in respiration medium (MiR05, outlined below) blotted on filter paper and weighed using a microbalance (Mettler-Toledo). Respiration of fiber bundles was then measured in mitochondrial respiration medium (MiR05) containing EGTA (0.5 mM), MgCl_2_.6H_2_O (3 mM), K-lactobionate (60 mM), taurine (20 mM), KH_2_PO_4_ (10 mM), HEPES (20 mM), sucrose (110 mM) and defatted BSA (1g.L-1) at pH 7.4, using the substrate-uncoupler-inhibitor titration protocol described below. All assays were performed, in duplicate, using an Oxygraph O2K (Oroboros Instruments, Innsbruck), with oxygen concentration kept between 250 to 400 pmol to prevent diffusion limitation of respiration. Respirometry was performed by the same operator throughout the study.

### 5.5 Substrate uncoupler inhibitor titration (SUIT) protocol

Oxygen flux (JO_2_) of the permeabilized muscle fibers was measured in the presence of a specific sequence of substrates, uncouplers and inhibitors, to quantify the maximum capacity of different components of mitochondrial respiration. The sequence of chemicals used to simulate the different respiratory capacities is summarized in Supplementary Table 3 and a representative trace of skeletal muscle fiber JO_2_ measured during the protocol is illustrated in Supplementary Fig. 1. Malate (2 mM, Sigma M1000) and octanoyl carnitine (0.2 mM, Tocris 0605) were initially added to the respiration medium to simulate the leak-limited respiration (LEAK). This leak-limited respiration was supported by fatty acid oxidation substrates and referred to as LEAK_*FAO*_. Subsequent addition of ADP (10 mM, Sigma A2754), also at saturating concentration, generated JO_2_ which supported ATP synthesis, but limited by fatty acid oxidation, OXPHOS_*FAO*_. Addition of pyruvate (20 mM, P2256) and glutamate (10 mM, Sigma G1626) saturated electron entry to complex I (OXPHOS_*CI*_), and addition of succinate (20 mM, Sigma S2378) saturated convergent electron flow via complex I and II to the Q-junction (OXPHOS_*MAX*_). Cytochrome c (10 μM) addition was used as a quality control to confirm outer mitochondrial membrane integrity; all assays with an increase in oxygen consumption of >15% following cytochrome *c* addition were excluded from further analysis. Carbonyl cyanide-p-trifluoromethoxyphenylhydrazone (FCCP) was used (stepwise titration of 0.5 μM, Sigma C2920) to uncouple oxidative phosphorylation and investigate ETS capacity (ETS_*MAX*_). Finally, rotenone was added (0.5 μM, Sigma C2920) to inhibit complex I [and thus FAO] and isolate succinate-linked ETS capacity (ETS_*CII*_). The OXPHOS coupling efficiency was calculated as follows to give an indication of mitochondrial coupling: OXPHOS coupling efficiency = 1 – LEAK_*FAO*_/OXPHOS_*MAX*_. The maximum respiratory capacities (OXPHOS_*MAX*_ and ETS_*MAX*_ were expressed per mg wet weight of muscle tissue. Individual capacities were expressed relative to ETS_*MAX*_, to indicate their proportional contribution.

### 5.6 Enzyme activity assays

Enzyme activity assays were performed as described previously [63]. Briefly, ~10 mg of vastus lateralis from each individual was homogenized with an Eppendorf pestle in an Eppendorf tube containing 300 μL of homogenization buffer containing Hepes (20 mM), EDTA (1 mM), and Triton X-100 (0.1% vol/vol). The samples were then centrifuged (380×*g*, 30 s, 4 °C), and the supernatant was collected. This supernatant was centrifuged again (380×*g*, 30 s, 4 °C), and the resulting supernatant was collected to obtain a homogeneous suspension. All assays were performed using a spectrophotometer (Evolution 220; Thermo Scientific) at 37 °C in a reaction volume of 1 mL. Citrate synthase activity was quantified with homogenate diluted to 10 μg of protein per mL in an assay buffer containing Tris (20 mM), 5,5′-dithiobis-2-nitrobenzoic acid (0.1 mM), and acetyl-CoA (0.3 mM) at pH 8.0. The reaction was initiated by the addition of oxaloacetate (0.5 mM), and the absorbance change at 412 nm was measured. The 3-hydroxyacyl-CoA dehydrogenase (HADH) activity was assayed with homogenate diluted to 20 μg of protein per mL in an assay buffer containing imidazole (50 mM), NADH (0.15 mM), and Triton X-100 (0.1% vol/vol) at pH 7.4. The reaction was initiated by the addition of 0.1 mM acetoacetyl-CoA (0.1 mM), and the absorbance change at 340 nm was measured. Enzyme activity values were corrected to the protein concentration of the homogenate measured using the Quick Start Bradford protein assay (Bio-Rad).

### 5.7 Mass spectrometry for skeletal muscle metabolomics

A chloroform/methanol extraction was performed on snap frozen skeletal muscle (~30 mg) as described previously [12], followed by ultra-high performance liquid chromatography mass spectrometry (UHPLC-MS) [64,65]. The aqueous phase was analyzed separately using normal and reverse phase analysis. The aqueous and organic fractions were then combined for carnitine analysis. The protein pellet was re-suspended in RIPA buffer (Thermo Scientific) containing protease inhibitor (Roche) and protein concentration determined using a bicinchoninic acid (BCA) assay (BCA1-1KT, Sigma). Data were processed using the Vendor software and normalized to protein concentration and to the intensity of internal standards.

### 5.8 Aqueous metabolite analysis

Reverse phase analysis was performed as described previously [65]. Before the analysis, samples were reconstituted in 0.1 mL of a 10 mM ammonium acetate water solution containing a mixture of 8 internal standards at the concentration of 10 μM (Proline, Valine D8, LeucineD10, Lys U13, Glutamic acid C13, Phen D5, Succinic acid D3, Serotonine D4). Normal phase analysis was performed using a Thermo scientific Vanquish™ UHPLC + series coupled with a TSQ Quantiva Triple Quadrupole Mass Spectrometer (Thermo Fisher Scientific, MA, United States) was used with an electrospray ionization (ESI) source, operated in positive and negative ion mode with polarity switching. The electrospray voltage was set to 3500 V for the positive ionization and to 2500 V for the negative ionization. Nitrogen at 48 mTorr and 420 °C was used as a drying gas for solvent evaporation. The aqueous phase was analyzed with a BEH Amide (150 × 2.1 mm 1.7 μm) column. The column was conditioned at 30 °C. The mobile phase consisted of: (A) a 0.1 M aqueous solution of ammonium carbonate and (B) acetonitrile. The mobile phase was pumped at a flow rate of 600 μL/min programmed as follows: initially stayed at 20% of A for 1.5 min, then was subjected to a linear increase from 20% to 60% of A in 2.5 min and kept at this percentage for 1 min and then brought back to initial condition after 0.1 min, followed by 3 min of equilibration.Xcalibur software (Thermo Fisher, version 4.1) was used for data acquisition. Putative recognition of all detected metabolites was performed using a targeted MS/MS analysis. Before the analysis, samples were reconstituted in 0.1 mL of an acetonitrile:1 M aqueous ammonium carbonate solution (7:3 v/v) containing a mixture of 3 internal standards at the concentration of 10 μM (Glutamic acid C13, Succinic acid D3 and AMP).

### 5.9 Carnitine analysis

Samples were prepared as described previously [12]. A UHPLC + series chromatography sstem coupled with a TSQ Quantiva Triple Quadrupole mass spectrometer (Thermo Fisher) was used with an ESI source, operating in positive and negative ionization mode at the same time. The electrospray voltage was set to 3500 V for the positive ionization and to 2500 V for the negative ionization. Nitrogen at 48 mTorr and 420 °C was used as a drying gas for solvent evaporation. The organic phases were analyzed with an ACE Excel 2C18 PFP (100A. 150 × 2.1 mm 5μ) column kept at 30 °C. The mobile phase consisted of: (A) a 0.1% of formic acid in water solution and (B) a methanol solution. The mobile phase was pumped at a flow rate of 0.450 μL/min programmed as follows: initially stayed at 0.5% of B for 1 min, then was subjected to a linear increase from to 100% of A in 9 min and kept at this percentage for 2 min and then brought back to initial condition after 0.1 min. Thermo Scientific Xcalibur software was used for data acquisition. Putative recognition of all detected metabolites was performed using a targeted MS/MS analysis. Before the analysis, samples were reconstituted in 0.1 mL of a methanol: water solution (4:1 v/v) containing a labelled carnitine standard set B mix solution (CN, C2, C3, C4, C5, C8, C14, C16).

### 5.10 Mass spectrometry for skeletal muscle and plasma lipidomics

#### 5.10.1 Sample preparation

A chloroform/methanol extraction was performed, as stated above for muscle, on 20 μl snap frozen plasma. For both skeletal muscle and plasma, the dried organic fraction was reconstituted in 50 μL of methanol/chloroform (1:1) and vortexed thoroughly. 10 μL of the sample was then diluted into 190 μL of isopropyl alcohol/acetonitrile/water (2:1:1) and briefly vortexed.

#### 5.10.2 Lipidomics analysis

An LTQ Orbitrap Elite Mass Spectrometer (Thermo Fisher Scientific) was used in positive and negative modes. Metabolites were ionized by heated electrospray before entering the spectrometer. The source temperature was set to 420 °C, and the capillary temperature to 380 °C. In positive mode, the spray voltage was set to 3.5 kV, while in negative it was 2.5 kV. Data were collected using the Fourier transform mass spectrometer (FTMS) analyzer. The resolution was set to 60,000 and the data was obtained in profile mode. The full scan was performed across an *m*/*z* range of 110–2000. For both modes, 5 μL of sample was injected onto a C18 CSH column, 2.1 × 50 mm (1.7 μM pore size) (Waters), which was held at 55 °C using an Ultimate 3000 UHPLC system (Thermo Fisher Scientific). The mobile phase comprise solvents A (acetonitrile/water 60:40) and B (acetonitrile/isopropanol 10:90), run through the column in a gradient (40% B, increased to 43% B after 0.8 min, 50% B at 0.9 min, 54% B at 4.8 min, 70% B at 4.9 min, 81% B at 5.8 min, raised to 99% B at 8 min for 0.5 min before returning to 40% for 1.5 min). Total run time was 10 min, with a flow rate of 0.500 μL/min. In positive mode, 10 mM ammonium formate was added to solvents A and B. In negative mode, 10 mM ammonium acetate was the solvent additive. Solvent additives were chosen based on current literature [66]. Before analysis, 250 μl internal standard (IS) mix was added to each sample. This was composed of deuterated standards sourced from Avanti Polar Lipids (C16-d31 Ceramide, 16:0-d31–18:1 PA,16:0-d31–18:1 PC, 16:0-d31–18:1 PE, 16:0-d31–18:1 PG, 16:0-d31–18:1 PI,14:0 PS-d54, and 16:0-d31 SM) and CDN Isotopes/QMX Laboratories (18:0-d6 CE, 15:0-d29 FA, 17:0-d33 FA, 20:0-d39 FA, 14:0-d29 LPC-d13, 45:0-d87 TG, 48:0-d83 TG, and 54:0-d105 TG). IS mix was made in 1:1 methanol chloroform, and each standard was at 2.5 μg/ml. For processing, spectra were converted to.mzML files using MSConvert (Proteowizard) for subsequent analysis. XCMS software within R was used to process data and identify peaks. Peaks were identified based on an approximate FWHM of 5 s and a signal to noise threshold of 5. To improve peak identification, peaks had to be present in a minimum of 25% of the samples. Peaks were annotated by accurate mass and retention time using an in-house R script. Peak intensity was normalized to internal standards and, in the instance of skeletal muscle tissue, to protein concentration.

#### 5.10.3 Plasma nitric oxide status and redox markers

Production, metabolism and bioavailability of nitric oxide (NO) were assessed in arterial and venous EDTA plasma by measurement of its oxidative breakdown products, nitrite and nitrate (using high pressure liquid chromatography), total nitrosated species (by gas phase chemiluminescence following reduction by triiodide) and cyclic GMP (by ELISA), as described in detail elsewhere [67,68]. Samples for NO metabolite analysis were pre-treated with N-ethylmaleimide (NEM), whilst those for other biomarkers were used immediately after thawing without further derivatization. For the former, frozen plasma aliquots were thawed in the presence of excess NEM in PBS to achieve a final concentration of 10 mM NEM. This was accomplished by adding a 100 mM NEM stock solution in 10 mM PBS (pH 7.40) at a 1:10 (v:v) ratio to a sample aliquot after thawing, followed by vortexing and 15 min incubation. Concentrations of total nitrosation products (RXNO) were measured by gas-phase chemiluminescence NO following reductive denitrosation using a septum sealed reaction chamber containing triiodide in glacial acetic acid constantly purged with N_2_. Samples were further incubated with acidic sulfanilamide to remove excess nitrite from NEM-treated EDTA plasma by reaction with acidic sulfanilamide prior to analysis, followed by injection of treated aliquots into the reaction chamber. The amount of NO liberated from low-molecular weight and protein nitroso-species were quantified by a gas-phase chemiluminescence analyser (CLD 77 am sp, EcoPhysics), as previously described [67,68]. Nitrate and nitrite concentrations were quantified using a dedicated high-performance liquid chromatography analyser (ENO20, Eicom) – ion chromatography equipped with an in-line Cd/Cu column for nitrate reduction and Griess diatozation coupling reaction following sample deproteinization by precipitation with methanol and centrifugation at 16000×*g* for 20 min as noted previously [68]. All glassware, collection tubes and pipettes rinsed with ultrapure water to reduce background contaminant levels of nitrite and nitrate. Concentrations of total free thiols were determined spectrophotometrically, using dithionitrobenzoic acid (DTNB) as described previously [69]. Plasma protein was quantified using the Coomassie (Bradford) Protein Assay Kit (ThermoScientific, Pierce Technology, Illinois, USA). Concentration of cGMP was quantified using a commercially available competitive ELISA immunoassay kit (R&D Systems, Abingdon, UK), and followed according to manufacturer's instructions. Plasma total antioxidant capacity was measured by the Ferric Reducing Activity of Plasma (FRAP) assay as previously described [70]. This assay is based on the principle that the reduction of ferric (Fe^3+^) to ferrous (Fe^2+^) ions at low pH results in the formation of an intense blue-colored ferrous-tripyridyltriazine complex with a maximum absorption at 593 nm. Sample FRAP was quantified by comparing the absorbance of the reacted sample at 593 nm with a standard curve of known concentration of ferrous ions. Plasma lipid peroxidation was evaluated by measuring the thiobarbituric acid reactive substances (TBARS) as described previously [71]. A breakdown product of lipid peroxides, malondialdehyde (MDA) within a biological sample reacts with thiobarbituric acid (TBA), at high temperature and acidic conditions to form a 1:2 MDA-TBA adduct, with maximum absorption at 532 nm. TBARS were quantified in plasma, following this reaction, by comparing the absorbance at 532 nm in the sample with a standard curve of solutions of known concentration of MDA. Plasma biomarker concentrations were quantified in a staggered fashion to ensure reproducible processing times. Daily calibrations with authentic standards were performed for all biochemical assays. Analyses were performed in triplicate, and values averaged except for nitrite, nitrate and total nitroso species, where only a single determination was performed due to sample volume limitations.

#### 5.10.4 Thiol redox status and glutathione persulfide concentrations in erythrocytes

Thiol redox status in erythrocytes was measured using ultra-high performance liquid chromatography tandem mass spectrometry (UPLC-MS/MS), as described in detail elsewhere [72]. Briefly, reduced and oxidized glutathione, cysteine and homocysteine as well as sulfide were quantified following hypotonic lysis of frozen erythrocyte pellets; lysis was accomplished by addition of ice-cold 1 mM ammonium phosphate buffer pH 7.40 containing 10 mM *N*-ethylmaleimide (NEM)/2 mM EDTA at a 1:4 (v:v) ratio. NEM alkylates free sulfhydryl groups, preventing artificial autoxidation during the thawing process and enhancing ionization efficiency. Derivatized samples were spiked with internal standards, subjected to ultrafiltration for protein removal and injected onto UPLC-MS/MS. The same mass spectrometry platform was used for measurement of inorganic (S_2_^2−^) and organic (cysteine and glutathione-SSH) persulfides. Erythrocytes were lysed by addition to the frozen pellet of ice-cold MQ water supplemented with 10 mM iodoacetamide (IAM) and 1 mM tyrosine at a 1:4 (v:v) ratio. IAM is used to derivatize persulfide [73], while tyrosine stabilises polysulfide adducts [74]. Samples were subjected to ultrafiltration and injected onto UPLC-MS/MS. Sulfide and persulfide was analyzed using the chromatography and multiple reaction monitoring conditions previously described [73]. Glutathione, cysteine, glutathione persulfide and cysteine persulfide were identified using the mass transitions of *m*/*z* 365.0 → 235.5 for GS-IAM, *m*/*z* 397.0 → 267.5 for GS-S-IAM and *m*/*z* 178.8 → 90 for CyS-IAM and *m*/*z* 210.8 → 90 for CysSS-IAM, respectively; retention times were 1.35, 1.49, 1.75, 2.11, 2.45 and 2.52 for CyS-IAM, CySS-IAM, GS-IAM, S-(IAM)_2_, GSS-IAM and SS-(IAM)_2_, respectively. No cysteine persulfide was detected in erythrocytes. Whereas erythrocytic glutathione concentrations measured by the IAM method and the NEM method were almost identical, persulfide concentrations are estimates only due to the lack of stable-isotope labelled standards.

#### 5.10.5 Statistics

Given the paucity of deep phenotyping studies in critically ill patients in the published literature, in particular, using the multifaceted approach planned in this investigation, it was not possible to perform a single statistical assessment to ensure adequate power to detect differences between the groups, using all of the assays. The approach chosen to determine a minimum sample size, was to base the calculation on the most important variables of interest. Given that this was a study of potential bioenergetic adaptation, the capacity for respiration supported by proton leak (later defined as LEAK_*FAO*_) and fatty acid oxidation (OXPHOS_*FAO*_), and the specific activity of the HADH enzyme (representing the capacity of the FAO metabolic pathway) were chosen to represent the three most important aspects of a putative bioenergetic and metabolic phenotype in critical illness. “Normal” values for these variables, in a cohort of healthy volunteers at sea level, were previously determined by our own research group, using identical assays and protocols to those in this study [12]. The distributions of these variables were also determined in a population of Himalayan Sherpas, taken to represent an important phenotype of bioenergetic-metabolic adaptation to hypoxic stress. The differences in these variables demonstrated in Sherpas compared to lowlanders may have (i) whole-body functional significance, as Sherpas demonstrate enhanced hypoxic tolerance, and (ii) mechanistic plausibility, as they theoretically increase the efficiency of cellular energy production. Sample size calculations were performed using these values (taken from the data repository for this study and an online power calculation tool (https://clincalc.com/stats/samplesize.aspx) was used, based on two independent samples and a continuous endpoint. An alpha value of 0.05 was chosen (representing a 5% probability of a type I error; the false positive rate, or probability of detecting a difference between the critically ill and reference groups when it does not exist); and power of 80% was selected, as a standard threshold typically considered to represent an acceptable risk of type II error (or false negative rate) in clinical studies. Assuming an enrolment ratio of 1:1, the minimum sample sizes for the three assays are summarized in Supplementary Table 4. A greater degree of variation was expected in the critically ill cohort than these cohorts (given the variation in expected stress, age, comorbidity and medications), and so these sample sizes were used to represent a minimum target for recruitment. Recruitment of critically ill patients was prioritised over reference patients, at an enrolment ratio of 2:1, in order to allow exploration of differences between survivors and non-survivors in the critically ill group.

All data were tested for normality using the D'Agostino and Pearson omnibus normality test. Parametric continuous data were described using mean and 95% confidence intervals; non-parametric data using median and interquartile range. Frequency counts and percentages were used to describe categorical variables. The critically ill and healthy reference cohorts were compared using the Mann-Whitney test for independent data with non-parametric distributions. Survivors and non-survivors were compared using the Mann-Whitney test. Bivariate correlations were assessed using Spearman's test. All statistical tests were two-tailed and p values of <0.05 were considered statistically significant. All statistics were performed using GraphPad Prism 8 software (GraphPad Software, Inc).

#### 5.10.6 Study approval

All protocols received approval from the National Health Service (NHS) Health Research Authority (HRA). The study of patients with organ failure was reviewed by Camden and Islington NHS Research Ethics Committee (IRAS ID 159977, REC reference 14/LO/1832) and the study of healthy reference patients was reviewed by the East of England Cambridge South Research and Development Department (IRAS ID 208220; REC reference 16/EE/0317). Protocols also received local site approval from the Royal Free Hospital Research and Development department. Participants with capacity gave written, informed consent. If participants lacked capacity, this was instead sought from their allocated consultee (personal or professional). When capacity was regained, participants were again approached for written, informed consent for their data to be included in the study.

## Author contributions

HTM performed experimental design, tissue procurement, data generation, data analysis and interpretation and manuscript preparation. KOB performed data generation, data analysis and interpretation and manuscript preparation. BF, MM, JW, BDM performed data generation and analysis. AT performed data generation. JLG performed data interpretation and intellectual contribution and reviewed manuscript. MPG and MGM provided intellectual contribution, experimental design and reviewed manuscript. MF and AJM performed experimental design, data interpretation and manuscript preparation. DSM conceived the study, designed experiments, interpreted data and prepared the manuscript.

## Declaration of competing interest

DSM has received honoraria for speaking and consultancy work from Siemens Healthineers and Edwards Lifesciences, and is a director of Oxygen Control Systems Ltd. MPWG serves on the medical advisory board of Sphere Medical Ltd. and is a director of Oxygen Control Systems Ltd. He has received honoraria for speaking and/or travel expenses from BOC Medical (Linde Group), Edwards Lifesciences, and Cortex GmBH. MGM holds a University Chair sponsored at UCL by Smiths Medical and is a paid consultant for Edwards Lifesciences and Baxter. He is a director of Oxygen Control Systems Ltd. All other authors declare that they have no conflicts of interests.
