# Supplementary material for: Divergent trajectories of cellular bioenergetics, intermediary metabolism and systemic redox status in survivors and non-survivors of critical illness
Source: Redox Biol. 2021 Feb 20;41:101907. doi: 10.1016/j.redox.2021.101907 (PMC7937570; doi:10.1016/j.redox.2021.101907)
Supplement: Multimedia component 1 [file mmc1.docx]

**Supplementary Figure 1. Respirometric assessment of permeabilized skeletal muscle.**


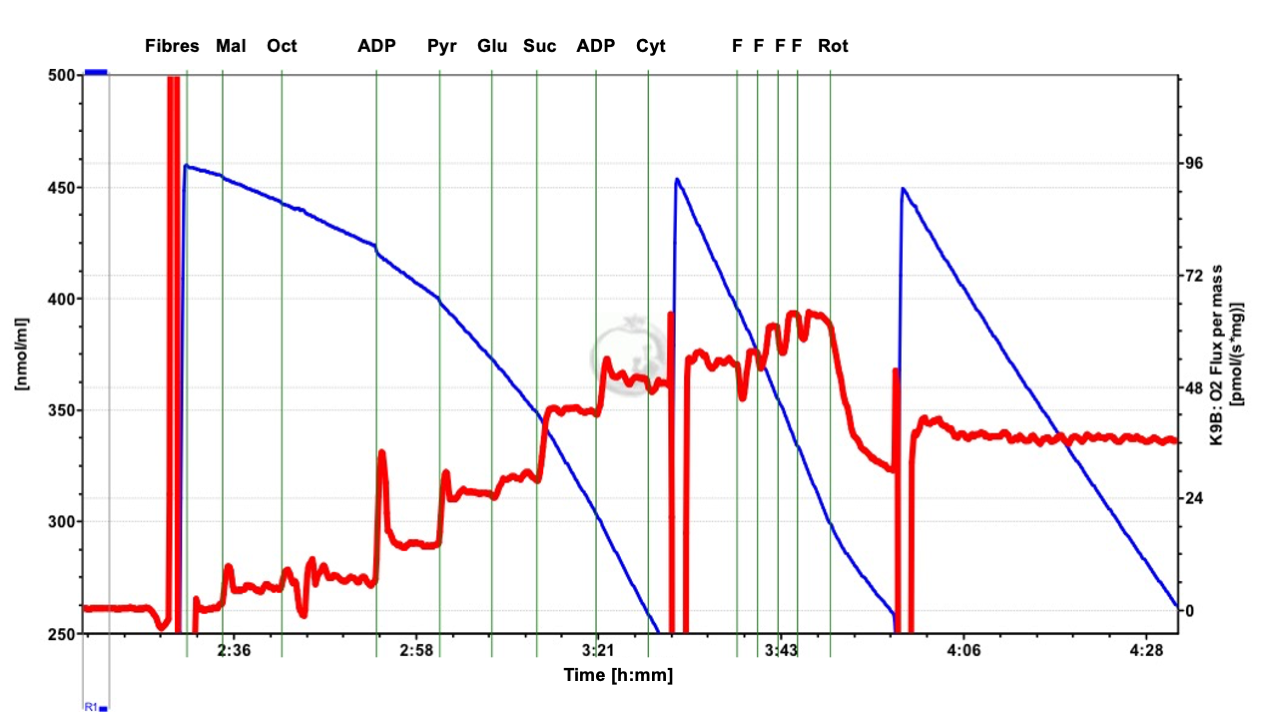


Representative Oxygraph trace generated by permeabilized muscle fibers during the substrate-uncoupler-inhibitor titration (SUIT) protocol, with the blue line indicating the oxygen concentration measured within the closed chamber (nmol/ml on left X-axis) and the red trace indicating the rate of oxygen consumption by the tissue within the chamber (J*_O2_*, right X-axis), and the green vertical markers indicating the addition of chemicals. Mal: malate; Oct: octanoyl carnitine; ADP; Pyr: pyruvate; Glu: glutamate; Suc: succinate; ADP; Cyt: cytochrome c; F: titrations of FCCP; Rot: rotenone. Volumes added to the respirometry chamber to give the desired final concentration are summarised in Supplementary Table 3.

**Supplementary Data Figure 2. Alterations in redox status of erythrocytes from venous blood of critically ill patients.**

Survivors (green circles; n=9 at < 48 h, 7 at Day 3-4, 6 at Day 5-7) and non-survivors (orange triangles; n=11 at < 48 h, 8 at Day 3-4, 8 at Day 5-7). Median (IQR). a, reduced glutathione (GSH) concentration. b, oxidised glutathione (GSSG). c, ratio of reduced to oxidised glutathione (GSH/GSSG). d, hydrogen sulfide (H2S). e, hydrogen persulfide (HS-SH). f, ratio of hydrogen sulfide to persufhide (H2S/HSSH). g, reduced cysteine. h, reduced homocysteine. i, glutathione persulfide (GS-SH). Mann-Whitney test, two-tailed. * p < 0.05

**Supplementary Data Figure 3. Inflammatory markers in survivors and non-survivors of critical illness.**


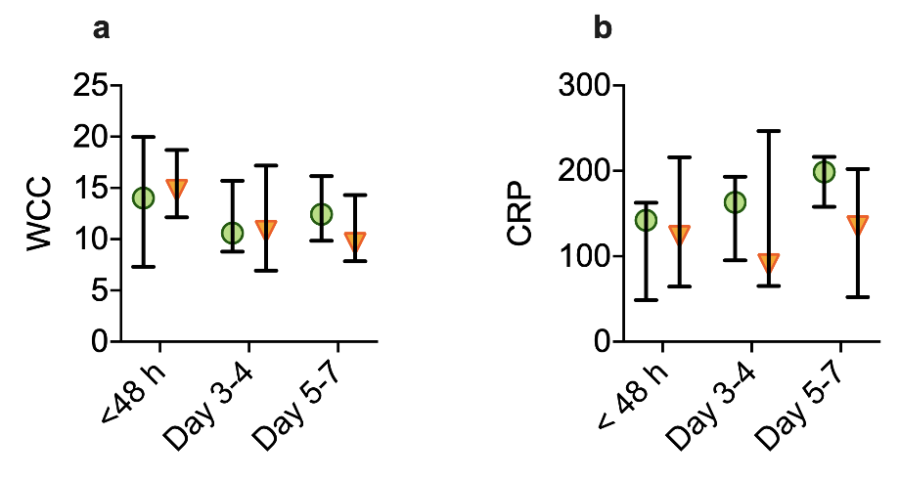


Survivors (green circles; n=9 at < 48 h, 7 at Day 3-4, 6 at Day 5-7) and non-survivors (orange triangles; n=12 at < 48 h, 9 at Day 3-4, 8 at Day 5-7). a, white cell count, b, C-reactive protein. Median (IQR). Mann-Whitney test, two-tailed. * p < 0.05

**Supplementary Table 1. Clinical characteristics of the critically ill cohort corresponding to the three timepoints at which biological samples were collected.**

| **Clinical characteristics at time of testing** | **Time after ICU admission** | | |
| --- | --- | --- | --- |
|  | **< 48 hours (n=21)** | **Day 3-4 (n=16)** | **Day 5-7**  **(n=14)** |
| SOFA score: median (IQR) | 12 (10.5-15) | 11.5 (6.5-14.8) | 9 (6.5-14.3) |
| Survival to hospital discharge: n (%) | 9 (43) | 7 (43) | 4 (33) |
| Mechanically ventilated: n (%) | 21 (100) | 13 (81) | 11 (79) |
| F_I_O_2_: % median (IQR) | 35 (30-45) | 38 (30-44) | 38 (30-46) |
| SpO_2_: % median (IQR) | 95 (94 -97) | 95 (93-97) | 96 (93-98) |
| PaO_2_: kPa median (IQR) | 11.6 (10.8-12.3) | 11.1 (10.0-13.2) | 11.5 (10.0-13.1) |
| CaO_2_: ml O_2_/100 ml blood median (IQR) | 14.6 (11.6-17.1) | 11.8 (10.8-14.8) | 12.0 (11.0-14.1) |
| Vasopressor requirement: mcg/kg/min median (IQR) | 0.19 (0.07-0.29) | 0 (0-0.14) | 0 (0-0.08) |
| Inotrope requirement: mcg/kg/min median (IQR) | 0 (0-0.04) | 0 (0-0) | 0 (0-0.81) |
| Mean arterial pressure: mmHg median (IQR) | 77 (73-81) | 83 (80-87) | 81 (75-88) |
| Heart rate: beats per minute median (IQR) | 80 (69-100) | 83 (68-110) | 93 (83-106) |
| Arterial lactate > 2.5 mmol/L: n (%)* | 6 (29) | 3 (19) | 2 (14) |
| Arterial lactate concentration: mmol/L median (IQR) | 1.6 (1.3-3.5) | 1.4 (0.9- 2.0) | 1.4 (0.9-1.8) |
| Renal replacement therapy: n (%) | 5 (24) | 4 (25) | 4 (29) |
| Estimated creatinine clearance: ml/min median (IQR)^#^ | 85 (39-116) | 100 (79-160) | 108 (91-139) |
| Serum albumin: g/L median (IQR) | 28 (22-35) | 27 (23-30) | 27 (24-30) |
| Serum bilirubin: μmol/L median (IQR) | 10 (5-18) | 12 (6-31) | 10 (6-19) |
| Serum ALT: units/L median (IQR) | 103 (34-270) | 97 (41-1043) | 51 (30-191) |
| Serum AST: units/L median (IQR) | 83 (48-315) | 79 (43-320) | 108 (91-139) |
| Temperature: °C median (IQR) | 36.8 (36.0-37.1) | 36.9 (36.6-37.2) | 36.8 (36.6-37.2) |
| White cell count: x10^9^/L median (IQR) | 14 (10.9-18.9) | 10.7 (7.9-15) | 10.3 (8.8-14.6) |
| C-reactive protein: mg/L median (IQR) | 126 (64-190) | 147 (72-190) | 181 (87-215) |
| Intravenous antibiotic therapy: n (%) | 19 (90) | 16 (100) | 14 (100) |
| Steroid therapy in previous 24 hours: n (%) | 11 (52) | 10 (63) | 7 (50) |
| Total steroid dose in 24 hours:  mg hydrocortisone equivalents median (IQR) | 30 (0-175) | 100 (0-200) | 160 (100-240) |
| Intravenous sedation: n (%) | 20 (95) | 10 (63) | 8 (57) |
| Continuous propofol infusion: n (%) | 11 (52) | 0 (0) | 1 (7) |
| Total propofol dose in previous 24 h: mg median (IQR) | 495 (0-2365) | 0 (0-0) | 0 (0-0) |
| Continuous fentanyl infusion: n (%) | 20 (95) | 11 (69) | 7 (50) |
| Continuous midazolam: infusion n (%) | 12 (57) | 4 (25) | 4 (29) |
| Receiving nutrition at time of biopsy: n (%) | 13 (62) | 14 (87) | 14 (100) |
| Enteral nutrition: n (%) | 12 (57) | 10 (63) | 9 (64) |
| Parenteral nutrition: n (%) | 1 (5) | 4 (25) | 5 (36) |
| Protein administered: g/24 hours median (IQR) | 5 (0-28) | 72 (20-83) | 75 (75-98) |
| Carbohydrate administered: g/24 hours median (IQR) | 23 (0-103) | 187 (94-225) | 187 (187-223) |
| Fat administered: g/24 hours median (IQR) | 4 (0-22) | 56 (15-61) | 56 (55-61) |
| Energy administered: kcal/24 hours median (IQR) | 160 (0-1653) | 1580 (510-1689) | 1600 (1580-1816) |
| Muscle relaxant within previous 24 hours: n (%) | 9 (43) | 0 (0) | 0 (0) |
| Continuous nitrate infusion: n (%) | 0 (0) | 0 (0) | 0 (0) |
| Blood glucose: mmol/L median (IQR) | 8 (6-10) | 9 (7-10) | 8 (8-9) |
| Rate of continuous insulin administered in previous hour: iu/h median (IQR) | 0 (0-2.5) | 0 (0-2) | 0 (0-10) |

SOFA: Sequential Organ Failure Assessment (range 0-24) (1); F_I_O_2_: Fractional concentration of inspired oxygen; SpO_2_: arterial haemoglobin percentage oxygen saturation determined by pulse oximetry; PaO_2_: partial pressure of oxygen dissolved in arterial blood, CaO_2_: arterial oxygen content, ALT: alanine aminotransferase; AST: aspartate aminotransferase.

* best threshold to predict acute mortality in patients with sepsis-induced organ failure (2).

^#^ indicates measurements in patients not undergoing renal replacement therapy.

**Supplementary Table 2. Baseline characteristics of survivors and non-survivors of critical illness.**

| Baseline and clinical characteristics at < 48 h | Survivors (n=9) | Non-survivors (n=12) |
| --- | --- | --- |
| Age: y mean (95% CI) | 55.3 (45.5-65.2) | 70.5 (51.5-77.8) |
| Female sex: n (%) | 2 (22) | 6 (50) |
| Height, m median (IQR) | 1.75 (1.69-1.81) | 1.73 (1.64-1.77) |
| Weight: kg median (IQR) | 75 (60.8-94.5) | 70.3 (63-90) |
| BMI: kg/m^2^ median (IQR) | 24.2 (21.4-34.3) | 25.3 (21.7 - 29.5) |
| Severe organ insufficiency prior to admission: n (%) | 1 (11) | 2 (16) |
| Independent prior to hospital admission: n (%) | 9 (100) | 11 (92) |
| Primary pathology: n (%) Infection Hemorrhage Coronary ischemia/arrhythmia/cardiogenic shock Respiratory pathology Acute liver impairment Acute renal impairment Gastrointestinal / intra-abdominal pathology | 6 (67) 1 (11) 2 (22) 5 (56) 2 (22) 1 (11) 5 (56) | 8 (67) 2 (17) 6 (50) 5 (42) 0 (0) 2 (16) 3 (25) |
| APACHE II score: median (IQR) | 23 (19-27) | 30.5 (29-37) |
| Admission SOFA score: median (IQR) | 11 (10-12) | 15 (11-17) |
| F_I_O_2_: % median (IQR) | 30 (30-43) | 40 (30-49) |
| SpO_2_: % median (IQR) | 96 (95-98) | 95 (94-96) |
| PaO_2_: kPa median (IQR) | 11.6 (10.8-14.1) | 11.6 (10.3-12.0) |
| CaO_2_: (ml/100 ml blood) median (IQR) | 15.3 (11.7-17.5) | 13.9 (11.4-16.7) |
| Vasopressor requirement: mcg/kg/min median (IQR) | 0.19 (0.05-0.24) | 0.25 (0.08-0.52) |
| Inotrope requirement: mcg/kg/min median (IQR) | 0 (0-0) | 0 (0-0) |
| Mean arterial pressure: mmHg median (IQR) | 77 (75-81) | 77 (72-83) |
| Arterial lactate: > 2.5 mmol/L n (%)* | 1 (11) | 5 (42) |
| Arterial lactate: mmol/L median (IQR) | 1.4 (1.3-1.5) | 2.1 (1.7-4.9) |
| Renal replacement therapy: n (%) | 1 (11) | 4 (33) |
| Estimated creatinine clearance: ml/min median (IQR) # | 103 (74-136) | 43 (20-103) |
| Serum albumin: g/L median (IQR) | 27 (19-38) | 30 (24-35) |
| Serum bilirubin: μmol/L median (IQR) | 10 (4-14) | 14 (8-39) |
| Serum ALT: units/L median (IQR) | 31 (19-115) | 138 (86-575) |
| Serum AST: units/L median (IQR) | 41 (20-140) | 126 (69-857) |
| Temperature: °C median (IQR) | 37.1 (36.6-37.6) | 36.6 (35.8-36.8) |
| White cell count: x 10^9^/L median (IQR) | 14 (7-20) | 14.8 (12.1-18.7) |
| C-reactive protein: mg/L median (IQR) | 142 (49-163) | 123 (64-216) |
| Intravenous antibiotic therapy: n (%) | 8 (89) | 11 (92) |
| Steroid therapy in previous 24 h: n (%) | 5 (56) | 6 (50) |
| Total steroid dose in 24 h: mg hydrocortisone equivalents median (IQR) | 100 (0-160) | 50 (0-188) |
| Intravenous sedation: n (%) | 9 (100) | 11 (92) |
| Continuous propofol infusion: n (%) | 7 (78) | 4 (33) |
| Total propofol dose in previous 24 h: mg median (IQR) | 1280 (210-2440) | 25 (0-2300) |
| Continuous fentanyl infusion: n (%) | 9 (100) | 11 (92) |
| Continuous midazolam infusion: n (%) | 4 (44) | 8 (67) |
| Receiving nutrition at time of biopsy: n (%) | 6 (66) | 7 (58) |
| Enteral nutrition: n (%) | 5 (56) | 7 (58) |
| Parenteral nutrition: n (%) | 1 (11) | 1 (8) |
| Protein administered: g/24 h median (IQR) | 8.9 (0-39) | 2 (0-30) |
| Carbohydrate administered: g/24 h median (IQR) | 29 (0-117) | 13 (0-103) |
| Fat administered: g/24 h median (IQR) | 0.1 (0-0.4) | 0.03 (0-0.4) |
| Energy administered: kcal/24 h median (IQR) | 200 (0-870) | 90 (0-686) |
| Muscle relaxant within previous 24 h: n (%) | 4 (44) | 5 (42) |
| Continuous nitrate infusion: n (%) | 0 (0) | 0 (0) |
| Blood glucose: mmol/L median (IQR) | 7 (6-11) | 8 (6-10) |
| Rate of continuous insulin administered in previous hour: iu/h median (IQR) | 0 (0-7) | 0 (0-2.5) |

BMI: Body mass index; APACHE II: Acute Physiology and Chronic Health Score (range 0-100) (1); SOFA: Sequential Organ Failure Assessment range 0-24) (3); F_I_O_2_: Fractional concentration of inspired oxygen; SpO_2_: arterial oxygen saturation determined by pulse oximetry; PaO_2_: partial pressure of oxygen dissolved in arterial blood, CaO_2_: arterial oxygen content; ALT: alanine aminotransferase; AST: aspartate aminotransferase.

* best threshold to predict acute mortality in patients with sepsis-induced organ failure (2).

# indicates measurements in patients not undergoing renal replacement therapy.

**Supplementary Table 3. Respirometric assessment of permeabilized skeletal muscle fibers. Substrate uncoupler inhibitor titration protocol used to simulate different respiratory capacities.**

| **Respiratory capacity** | **Isolation of respiratory pathway supporting oxygen flux** | **Reagents added to chamber** |
| --- | --- | --- |
| LEAK*_FAO_* | Proton leak from intermembrane space to matrix, supported by electron entry via fatty acid oxidation, but in the absence of ADP | Malate, 2 mM Octanoyl carnitine, 0.2 mM |
|  |  |  |
| OXPHOS*_FAO_* | Oxidative phosphorylation supported by electron entry via fatty acid oxidation | ADP, 10 mM |
| OXPHOS*_CI_* | Oxidative phosphorylation supported by saturating electron entry at complex I | Pyruvate, 20 mM Glutamate, 10 mM |
|  |  |  |
| OXPHOS*_MAX_* | Oxidative phosphorylation supported by convergent electron flow to the Q junction via complexes I and II | Succinate, 10 mM (further addition of ADP to ensure saturation) |
|  |  |  |
| Quality control | Test for mitochondrial outer membrane integrity  Data discarded if > 15% increase in JO2 | Cytochrome c, 10 μM |
| ETS*_MAX_* | Electron transfer system uncoupled from ADP phosphorylation, supported by convergent electron flow to the Q junction via complexes I and II. | FCCP  (stepwise titration 0.5 μM until inhibitory effect observed) |
| ETS*_CII_* | Electron transfer system, uncoupled from ADP phosphorylation, supported by electron entry via complex II, following inhibition of complex I | Rotenone, 0.5 μM |

**Supplementary Table 4. Sample size estimation for three important aspects of bioenergetic/metabolic phenotype**

| **Critical variables** | **Anticipated values for reference cohort*** | | **Threshold of difference to detect in critically ill cohort^#^** | **Sample size** |
| --- | --- | --- | --- | --- |
|  | Mean | Standard deviation | Mean |  |
| **LEAK*_FAO_* (pmol/s/mg)** | 10.1 | 1.77 | 7.04 | 10  (5 per group) |
| **OXPHOS*_FAO_* (pmol/s/mg)** | 23.6 | 7.13 | 15.6 | 26  (13 per group) |
| **HADH activity**  **μmol/min/mg** | 0.39 | 0.09 | 0.28 | 24  (12 per group) |

*based on values for healthy lowlanders at sea level (4)

**^#^**based on values for Sherpas (representing putative adaptive phenotype) (4).

**Supplementary References**

1. Knaus WA, Draper EA, Wagner DP, Zimmerman JE. APACHE II: a severity of disease classification system. Crit Care Med. 1985;13:818-829.

2. Filho RR, Rocha LL, Corrêa TD, Pessoa CM, Colombo G, Assuncao MS. Blood Lactate Levels Cutoff and Mortality Prediction in Sepsis-Time for a Reappraisal? a Retrospective Cohort Study. Shock. 2016;46:480-485.

3. Moreno R, Vincent JL, Matos R et al. The use of maximum SOFA score to quantify organ dysfunction/failure in intensive care. Results of a prospective, multicentre study. Working Group on Sepsis related Problems of the ESICM. Intensive Care Med. 1999;25:686-696.

4. Horscroft JA, Kotwica AO, Laner V et al. Metabolic basis to Sherpa altitude adaptation. Proc Natl Acad Sci U S A. 2017;114:6382-6387.
